# Norepinephrine changes behavioral state through astroglial purinergic signaling

**DOI:** 10.1126/science.adq5233

**Published:** 2025-05-15

**Authors:** Alex B. Chen, Marc Duque, Altyn Rymbek, Mahalakshmi Dhanasekar, Vickie M. Wang, Xuelong Mi, Loeva Tocquer, Sujatha Narayan, Emmanuel Marquez Legorreta, Mark Eddison, Guoqiang Yu, Claire Wyart, David Prober, Florian Engert, Misha B. Ahrens

**Affiliations:** 1 Janelia Research Campus, Howard Hughes Medical Institute; Ashburn, VA 20147, USA; 2 Department of Molecular and Cellular Biology, Harvard University; Cambridge, MA 02138, USA; 3 Graduate Program in Neuroscience, Harvard Medical School; Boston, MA 02115, USA; 4 Tianqiao and Chrissy Chen Institute for Neuroscience, Division of Biology and Biological Engineering, California Institute of Technology, Pasadena, CA, 91125, USA; 5 Sorbonne Université, Paris Brain Institute (Institut du Cerveau, ICM), Institut National de la Santé et de la Recherche Médicale U1127, Centre National de la Recherche Scientifique Unité Mixte de Recherche 7225, Assistance Publique–Hôpitaux de Paris, Campus Hospitalier Pitié-Salpêtrière, Paris, France; 6 Bradley Department of Electrical and Computer Engineering; Virginia Polytechnic Institute and State University; Arlington, VA 22203, USA; 7 Present address: Allen Institute for Brain Science; Seattle, WA 98109, USA; 8 Department of Automation, Tsinghua University; Beijing 100084, P.R. China

## Abstract

Both neurons and glia communicate through diffusible neuromodulators; however, how neuron-glial interactions in such neuromodulatory networks influence circuit computation and behavior is unclear. During futility-induced behavioral transitions in the larval zebrafish, the neuromodulator norepinephrine (NE) drives fast excitation and delayed inhibition of behavior and circuit activity. We found that astroglial purinergic signaling implements the inhibitory arm of this motif. In larval zebrafish, NE triggers astroglial release of adenosine triphosphate (ATP), extracellular conversion of ATP into adenosine, and behavioral suppression through activation of hindbrain neuronal adenosine receptors. Our results suggest a computational and behavioral role for an evolutionarily conserved astroglial purinergic signaling axis in NE-mediated behavioral and brain state transitions and position astroglia as important effectors in neuromodulatory signaling.

Neural circuits perform fast computations through precise patterns of synaptic connectivity and direct electrical coupling through gap junctions (1, 2), but they can also be rapidly modulated by diffusible chemical messengers, including monoamines (such as norepinephrine, dopamine, serotonin) and neuropeptides (3–5). Such signaling accounts for a large portion of neural activity patterns that cannot be explained by synaptic connectivity alone (6–8) and has long been known to reconfigure synaptic networks to orchestrate behavioral states (9–13). Recent discoveries have shown that astroglia communicate bi-directionally with neurons through neuromodulatory signaling, suggesting that non-neuronal cells could play more important roles as neuromodulatory actuators than previously thought (14). Astrocytes and neurons possess substantially different physiologies. Astrocytes are electrically inexcitable, exhibit local and global intracellular calcium transients, and possess complex arbors of processes that form non-overlapping territories and interact with thousands of individual neuronal synapses (15, 16). However, how the unique physiology of astrocytes contributes to their role as active modulatory elements in neural circuits is still unresolved.

Our work focuses on two major neuromodulators, norepinephrine (NE) and adenosine triphosphate (ATP)/adenosine and their role in mediating behavioral state changes. Since its discovery in the 1940s (17), NE has been known to profoundly influence neurophysiology, neural circuit dynamics, and behavior (17–25). While the dominant assumption over the past eight decades has been that NE acts primarily via activation of adrenergic receptors on neurons, recent discoveries that NE also activates non-neuronal cells, particularly astroglia, challenge this assumption. In astroglia, NE triggers large intracellular calcium events caused by α1-adrenergic receptor (α1-AR) activation (26–30), but the specific roles played by astroglia in noradrenergic modulation remain unclear, as do the pathways linking NE-mediated astroglial calcium elevation to modulation of circuit activity.

As with NE, the purinergic signaling molecules ATP and adenosine are ubiquitous and critical neuromodulators. They play important roles in sleep-wake cycles (31–33), synaptic plasticity (34), and motor pattern generation (35), among a number of other functions (36–38). Although astroglia have been argued to be a source of extracellular adenosine through ATP secretion (34, 36) and extracellular ATP-to-adenosine metabolism (39), the behavioral relevance of such release remains, in many cases, controversial (40, 41). Furthermore, while astroglial calcium elevation appears to trigger ATP secretion, the behavioral contexts that recruit astroglial purinergic signaling remain poorly understood, due to the reliance on exogenous activation and/or ex vivo conditions in existing studies (42–44).

Leveraging the larval zebrafish, in which NE, ATP, and astroglial calcium can be imaged in conjunction with neural activity during behavior, we find that, during rapid behavioral state transitions, the noradrenergic and purinergic systems can be conceptualized as, respectively, fast excitatory and delayed inhibitory arms of a feedforward motif with astroglia as a coordinating intermediary. Therefore, beyond slow modulation of state, NE also acts through astroglial purinergic signaling to rapidly reconfigure circuit dynamics and enact behavioral state transitions.

## NE neurons drive a biphasic futility response

**Figure 1.**
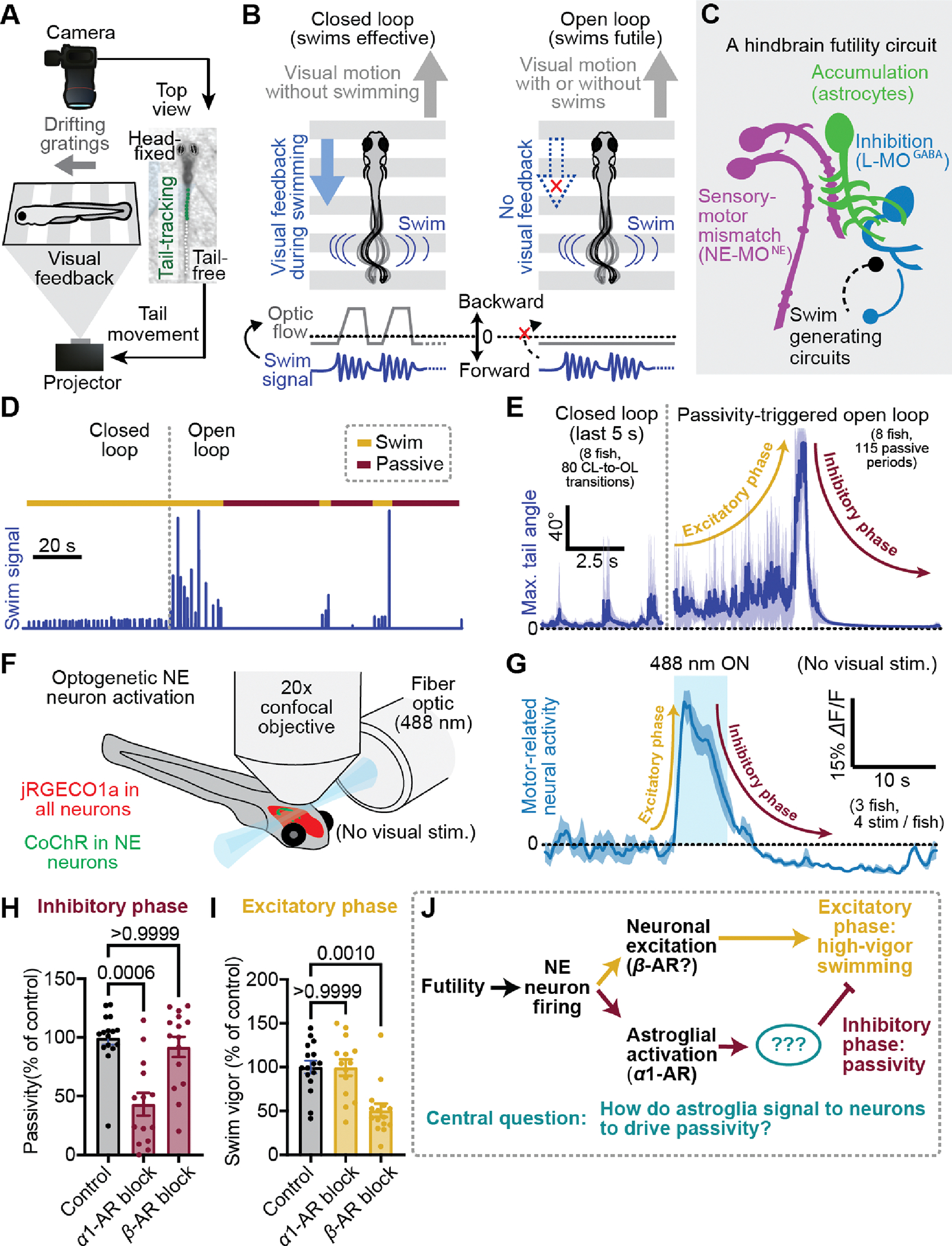
Futility triggers a biphasic behavioral and neural response through NE neuron activation. (**A**) Schematic of virtual reality behavioral experiments with real-time swim detection and visual feedback. (**B**) Diagram illustrating the difference between closed loop (visual feedback in response to swims) and open loop (no visual feedback) conditions. (**C**) Schematic: the known cell types involved in futility-induced passivity. (**D**) Swim trace of an example trial demonstrating closed and open loop swim behavior. (**E**) Average closed loop and passivity-triggered open loop tail angle demonstrating an initial increase in swim amplitude (excitatory phase) followed by inhibition of swimming (inhibitory phase) in open loop. (**F**) Neural activity, imaged with a confocal microscope while NE neurons were optogenetically activated using a fiber optic. (**G**) Optogenetic stimulation-triggered average of neural activity in motor areas demonstrating fast excitation and delayed inhibition, similar to the behavioral futility response. (**H,I**) Effect of blocking *α*1-adrenergic receptors (100 *μ*M prazosin) or *β*-adrenergic receptors (100 *μ*M propranolol) on (**H**) open loop passivity and (**I**) open-loop swim vigor. Panel H,I: N = 16 (control), 14 (α1-AR block), 15 (β-AR block) fish. Panel H: P > 0.9999 (control vs. β-AR block), P = 0.0006 (control vs. α1-AR block) Panel I: P = 0.001 (control vs. β-AR block), P > 0.9999 (control vs. α1-AR block). Kruskal-Wallis. (**J**) Model of parallel noradrenergic channels that contribute to the excitatory and inhibitory phases of the futility response and central problem statement. All error bars and shaded error regions represent s.e.m.

Larval zebrafish possess an innate tendency to stabilize their position by swimming in the direction of coherent visual flow (45, 46). We have previously shown that when swims no longer move the fish forward, futility drives firing in hindbrain NE neurons, and NE signals through radial astroglia, a glial cell type similar to mammalian astrocyte (47, 48), to suppress futile swims (49). Here we made use of our previously published behavioral assay for futility-induced passivity in larval zebrafish (49, 50). Fish were immobilized in agarose and their tails freed. The animals’ tail positions were then automatically tracked, and detected swims used to deliver realistic online visual feedback through projection of drifting grating stimuli to the floor of the chamber (Fig. 1A, Methods). To encourage robust swimming behavior, we delivered a steady, constant-velocity forward drifting grating (45) (Fig. 1B). Simultaneously, we manipulated the efficacy of the fish’s swims by cycling between two stimulus conditions: closed loop, and open loop. During closed loop, the fish’s swim attempts resulted in visual (backward drift of the visual stimuli) to signal successful forward swimming (Fig. 1B, left). In open loop, swim attempts resulted in no change to the visual stimulus (Fig. 1B, right) and are thus futile. Futility is encoded by a population of NE neurons in the medulla oblongata known as NE-MO (putatively homologous to mammalian cluster A2 (51)), and NE-MO activation drives astroglial calcium elevation to drive motor-inhibitory GABAergic neurons in the lateral medulla oblongata (L-MO) (Fig. 1C) (49).

Consistent with previous work (26, 49), we found that behavioral futility signaled by a lack of visual feedback caused fish to enter a passive state, in which swimming ceases for tens of seconds (Fig. 1D,E, fig. S1, Video S1). Prior to passivity, fish exhibited an increase in swim vigor and were more likely to perform high-amplitude, struggle-like swims (Fig. 1D,E, fig. S1). Although NE-MO activation was previously shown to cause passivity (49), the contribution of NE neurons in the transient upregulation of swim vigor at futility onset has not been thoroughly investigated. Given NE’s well-documented ability to enhance arousal and effort (21, 52), we tested whether NE neuron firing immediately promotes the rapid enhancement of vigor, in addition to driving temporally delayed swim inhibition (Fig. 1F, fig. S2). We found that optogenetic stimulation of NE neurons drove fast excitation and persistent, but delayed inhibition of hindbrain motor circuits (Fig. 1G, fig. S2A–C). We further observed that persistent inhibition of motor circuits coincided with sustained activity in L-MO (49) (fig. S2D). Therefore, in larval zebrafish, behavioral futility promotes a biphasic response in both behavior and neural dynamics, which can be defined as an excitatory phase, consisting of increased behavioral vigor, followed by an inhibitory phase involving behavioral suppression (Fig. 1E,G). Both phases are driven by NE neuron firing.

NE neurons act on downstream targets by activating alpha adrenergic receptors (α-ARs) and beta adrenergic receptors (β-ARs) by NE, as well as via fast synaptic excitation through co-released glutamate. Astroglial calcium elevation through α1-AR activation has been shown to be both necessary and sufficient for the inhibitory phase of the futility response in larval zebrafish (49), but its relationship to the excitatory phase of the futility response is less understood. Our findings suggest that astroglial calcium signaling is not involved in the excitatory phase: first, optogenetic stimulation of NE neurons elevated astroglial calcium with a temporal delay likely too long to account for the more rapid increase in motor activity (fig. S3A). Second, inhibition of α1-AR signaling with prazosin, which completely abolishes NE-evoked astroglial calcium responses (49), had no effect on futility-induced vigor enhancement, but did suppress futility-induced passivity (Fig. 1H,I). Thus, α1-AR signaling and astroglial calcium elevation are likely dispensable for the excitatory phase.

The activation of α1-ARs and downstream astroglial calcium elevation seem to act in a feedforward inhibitory-like manner Feedforward inhibition in synaptic networks serves to sharpen the window of excitatory drive (53, 54). Similarly, elevating astroglial calcium activation through optogenetic stimulation in Tg(gfap:CoChR-eGFP) fish or inhibiting calcium using prazosin shortened or lengthened the window of higher vigor open loop, respectively (fig. S3B,C). Furthermore, longer-duration NE-MO stimulation reliably triggered swim cessation within a few seconds (fig. S3D), despite NE likely remaining elevated for much longer (50), consistent with feedforward inhibition, rather than feedback inhibition, of noradrenergic drive.

On the other hand, blockade of β-ARs strongly attenuated the excitatory phase (Fig. 1H,I), suggesting that different adrenergic receptor subtypes may implement different aspects of the futility response. Blocking β-ARs also reduced passivity in some fish, potentially because inhibiting the excitatory phase reduces higher vigor struggles leading to less NE release (26, 49), lowering subsequent passivity. Alternatively, β-ARs could mediate feedback inhibition of the excitatory phase. Nevertheless, specific astroglial involvement in the inhibitory phase raises a fundamental question, central to this work (Fig. 1J): as neurons ultimately control motor output, how do astroglia signal to downstream neurons to drive the inhibitory phase by suppressing swimming?

## Futility-induced, NE-dependent astroglial ATP release

**Figure 2.**
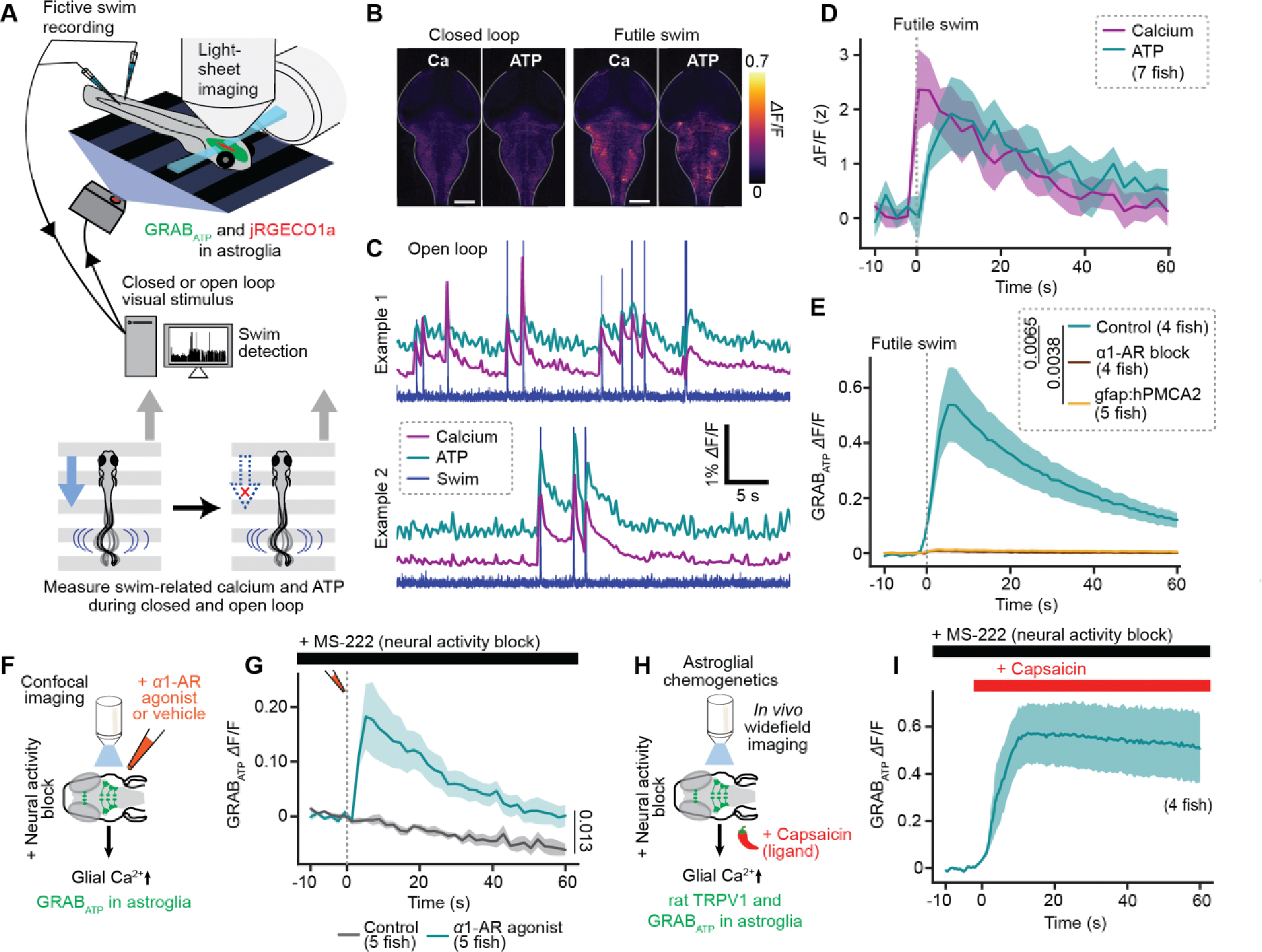
Futility drives astroglial release of ATP. (**A**) Experimental schematic: two-color light-sheet imaging of extracellular ATP and astroglial calcium (*Tg(gfap:GRAB*_*ATP*_; *gfap:jRGECO1a*) fish) along with fictive behavioral recording. (**B**) Fluorescence micrographs of simultaneously collected GRAB_ATP_ and jRGECO1a signals in a fish in baseline condition or during a futile swim. (**C**) Two examples of motor nerve electrical activity, GRAB_ATP_ and jRGECO1a signals during open loop periods. Swim, calcium, and ATP traces are manually offset along the vertical axis to allow for better visualization. (**D**) Futile-swim triggered astroglial calcium and extracellular ATP signals averaged across fish (N = 7). (**E**) Futile swim-triggered GRAB_ATP_ signal in fish treated with an *α*1-AR blocker (100 *μ*M prazosin) or vehicle, and in fish expressing hPMCA2 in astroglia. N = 4 (control), 5 (α1-AR block), 5 (hPMCA2). P = 0.0065 (control vs. α1-AR block), 0.0038 (control vs. hPMCA2). Kruskal-Wallis test on AUC from 0 to 60s. (**F**) Experimental schematic: *ex vivo* confocal imaging during puffing of an *α*1-AR agonist (10 *μ*M methoxamine) or vehicle in the presence of a neural activity blocker (160 mg/L MS-222, a sodium channel inhibitor). *Tg(gfap:GRAB*_*ATP*_*)* fish. (**G**) GRAB_ATP_ signal in fish in experiments described in (F), triggered on puff and onset-aligned. N = 5, both conditions. P = 0.013, Mann-Whitney on AUC from 0 to 60 s. (**H**) Experimental schematic: *in vivo* widefield imaging during chemogenetic activation of *Tg(gfap:rTRPV1-eGFP)* fish with 200 nM capsaicin in the presence of a neural activity blocker (170 mg/L MS-222). (**I**) GRAB_ATP_ signal in fish treated with capsaicin as described in (F), triggered capsaicin administration and onset-aligned. All error bars and shaded error regions represent s.e.m.

We reasoned that astroglia likely communicate with downstream neurons by secreting a neuroactive substance. In particular, we hypothesized that futility drives calcium-dependent astroglial release of ATP, as glial-derived ATP can modulate neural activity in other contexts (43, 44, 55, 56). To investigate whether futility-induced astroglial calcium elevation leads to release of ATP, we generated a fish line (Tg(gfap:GRABATP;gfap:jRGECO1a)) expressing GRABATP, an extracellular green fluorescent ATP sensor (57), as well as the intracellular red fluorescent calcium sensor jREGCO1 in astroglia (Methods). First, we validated GRABATP’s specificity for ATP over adenosine in fish (fig. S4A). We then performed simultaneous brain-wide functional imaging of both glial calcium and secreted ATP while immobilized animals behaved in virtual reality (Methods) (Fig. 2A). We found that, during open-loop swimming, both astroglial intracellular calcium and extracellular ATP around astroglia exhibited a rapid elevation throughout the hindbrain, followed by a slower return to baseline over tens of seconds (Fig. 2B–D).

These data suggest that astroglia, and not neurons, release ATP during futile swimming. However, since the ATP sensor used is extracellular, it cannot distinguish between astroglial-secreted ATP and ATP released by neurons near astroglial processes. However, five additional lines of evidence support an astroglial origin for the released ATP. First, ATP elevation lags behind intracellular astroglial calcium elevation (fig. S4B–D), consistent with astroglial calcium elevation causing ATP release. Second, simultaneous imaging of neuronal calcium activity and extracellular ATP revealed that neuronal calcium elevation failed to reliably predict ATP release, whereas glial calcium events were always accompanied by ATP elevation (fig. S4E–G). Third, inhibition of astroglial calcium with pharmacological blockade of α1-ARs strongly attenuated both futility-triggered ATP elevation (Fig. 2E) and astroglial calcium (fig. S4H) but had no effect on closed loop ATP or astroglial calcium dynamics (fig. S4H). Because this pharmacological manipulation is non-specific, we also generated a fish expressing the calcium extruder hPMCA2 specifically in astroglia (Tg(gfap:hPMCA2-mCherry)). Astroglial-specific hPMCA2 expression inhibited astroglial calcium elevation during futile swims (fig. S5A) and, accordingly, decreased the duration of passivity in open loop (fig. S5B–E). This effect was not due to a decrease in general health of the animal or in the health of astroglia, as animals expressing hPMCA2 in astroglia behaved similarly to wild-type siblings (fig. S5F–H) and possessed astroglia that still exhibited microdomain calcium events (fig. S5I–K). Inhibiting glial calcium elevation with hPMCA2 also suppressed futile swim-evoked ATP elevation (Fig. 2E). Fourth, pharmacological activation of α1-ARs was sufficient to cause ATP elevation even when neural activity was inhibited with a sodium channel blocker (Fig. 2F,G). Fifth, direct chemogenetic activation of astroglia also elevated ATP when neural activity was inhibited (Fig. 2H,I). Neither pharmacological nor chemogenetic astroglial activation affected neuronal activity under conditions of sodium channel block (fig. S6A,B). These converging lines of evidence implicate norepinephrine-mediated astroglial, and not neuronal, calcium signaling as critical for extracellular ATP elevation during behavioral futility.

## ATP promotes passivity through extracellular metabolism into adenosine

**Figure 3.**
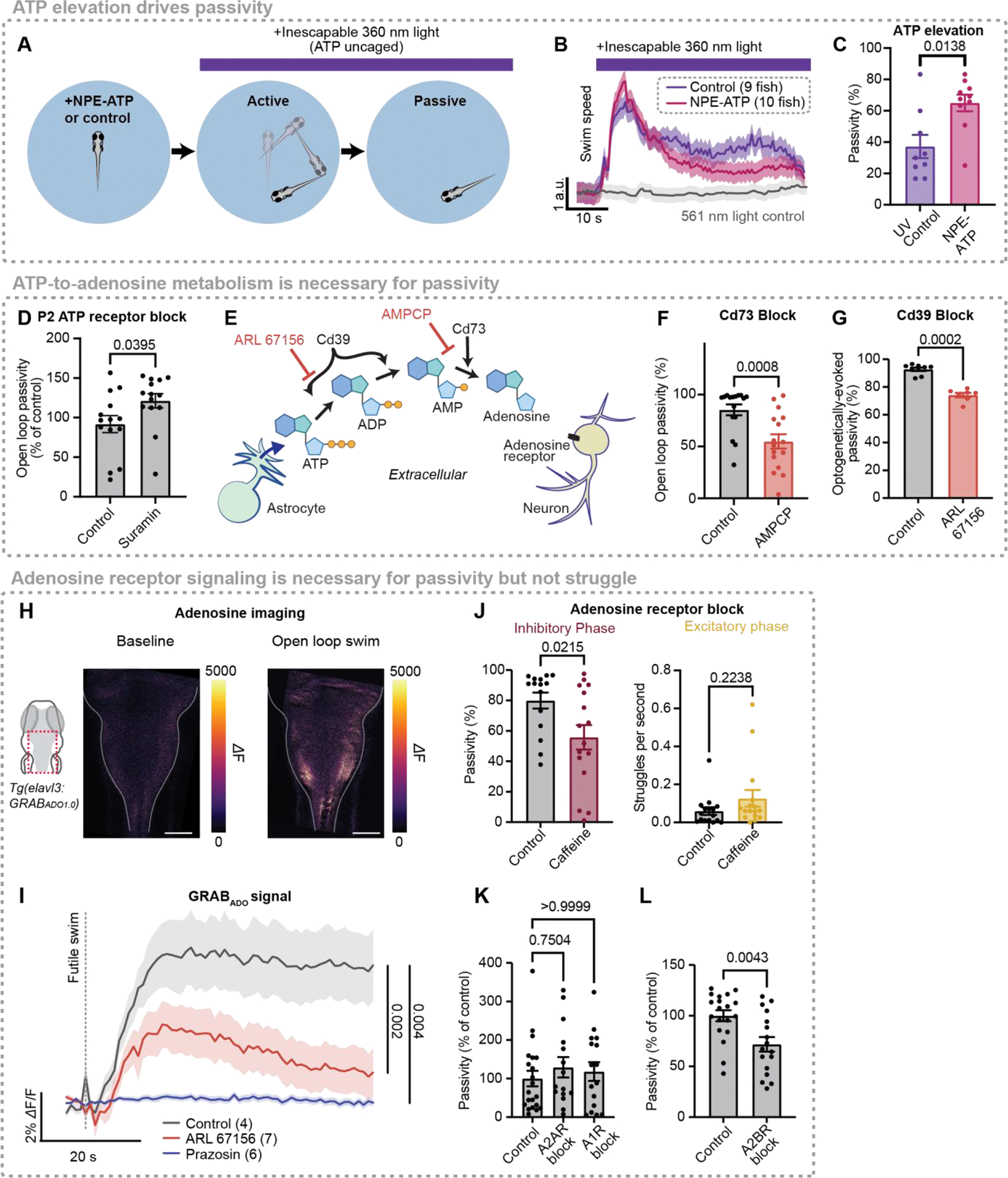
ATP promotes passivity via extracellular metabolism into adenosine. (**A**) Experimental schematic: Behavioral recording of freely swimming fish treated with 100 *μ*M NPE-ATP or vehicle then subjected to inescapable ultraviolet (360 nm) light, which uncages ATP. (**B**) Light onset-triggered swim speed of fish treated with NPE-ATP or vehicle. (**C**) Percent of light ON period spent passive for vehicle control and NPE-ATP treated fish. N = 9 (control), 10 (NPE-ATP). P = 0.0138, Mann-Whitney. (**D**) Effect of P2 receptor blocker (100 *μ*M suramin) or vehicle on open loop passivity in head-fixed behavior. N = 14 (control), 14 (suramin). P = 0.0395, Mann-Whitney. (**E**) Diagram illustrating extracellular biochemical ATP-to-adenosine pathway through enzymes Cd39 and Cd73, and pathway inhibition by competitive Cd39 inhibitor ARL 67156 and Cd73 inhibitor AMPCP. (**F**) Effect of Cd73 block (100 *μ*M AMPCP) on open loop passivity in head-fixed behavior. N = 16 (control), 16 (AMPCP). P = 0.0008, Mann-Whitney. (**G**) Percent passivity following onset of optogenetic stimulation for fish treated with ARL 67156 or vehicle. N = 9 (control), 7 (ARL 67156). P = 0.0002, Mann-Whitney. (**H**) Extracellular adenosine (GRAB_ADO1.0_) signal of a fish in baseline (left) and during a futile swim (right). (**I**) Futile-swim-triggered average of GRAB_ADO1.0_ signal in fish treated with vehicle, *α*1-AR blocker (100 *μ*M prazosin) or Cd39 inhibitor (1 mM ARL 67156). N = 4 (control), 7 (ARL 67156), 6 (prazosin); P = 0.002 (control vs. ARL), 0.004 (control vs. prazosin), Kruskal-Wallis on AUC from 0 – 60 s following futile swim. (**J**) Effect of vehicle or adenosine receptor blocker (100 *μ*M caffeine) on proportion of open loop spent passive (left) and open loop struggle rate (right). N = 15 (control), 16 (caffeine); P = 0.0215 (proportion passivity), 0.2238 (struggle rate), Mann-Whitney. (**K,L)** Effect of an A1R blocker (DPCPX) and an A2AR blocker (SCH-58261) (**K**) or an A2BR blocker (MRS 1754) (**L**) on proportion of open loop spent passive. Panel K: N = 21 (control), 15 (DPCPX), 16 (SCH-58261); N = 19 (control), 17 (MRS 1754). All error bars and shaded error regions represent s.e.m.

We investigated whether ATP elevation promotes passivity by treating fish with NPE-caged ATP (P(3)-[1-(2-nitrophenyl)]ethyl ester of ATP), which is pharmacologically inert until exposed to ultraviolet (UV) light (Methods) (Fig. 3A). Freely swimming fish treated with caged ATP or vehicle were exposed to UV light, and the time required for fish to switch from active swimming to passivity was recorded. Ultraviolet light constitutes an inescapable aversive stimulus, conceptually similar to the open loop conditions described in Figure 1. As a result, fish exposed to UV light eventually exhibited futility-induced passivity following a period of high-vigor swimming (Fig. 3B, fig. S7A). However, fish treated with caged ATP became passive more quickly than vehicle controls (Fig. 3B–C), while exhibiting no difference in struggle onset or time to peak swimming (fig. S7B). Therefore, ATP elevation drives the inhibitory, but not the excitatory, phase of futility-induced passivity.

Having established that ATP is an astroglia-to-neuron signal that induces passivity, we next sought to determine the mechanism through which ATP release suppresses swimming. ATP directly binds to two families of purinergic receptors (P2 receptors), ionotropic P2X receptors and metabotropic P2Y receptors. Surprisingly, broad P2 receptor inhibition with suramin (100 μM bath administration) did not inhibit futility-induced passivity (Fig. 3D). This result suggests that although ATP elevation drives passivity, P2 purinergic receptors do not mediate futility-induced swimming suppression.

Because direct action of ATP does not seem to play an important role in futility-induced passivity, we considered an alternative hypothesis, in which the ATP metabolite adenosine acts directly on neurons and suppresses swimming. Once secreted into the extracellular space, ATP is rapidly metabolized into adenosine through the action of two membrane-localized, extracellular-facing enzymes: Cd39 (or Entpd), which converts ATP into AMP by hydrolyzing the γ- and β-phosphate residues of ATP, and Cd73 (or Nt5e), which catabolizes AMP into adenosine (39) (Fig. 3E). In the spinal cord, such extracellular ATP-to-adenosine conversion is thought to contribute to locomotor rhythms (35). We performed two experiments to test the involvement of both components of this extracellular, biochemical pathway in futility-induced passivity. First, we blocked Cd73 with AMPCP and found that futility-induced passivity was inhibited (Fig. 3E,F). Additionally, we treated fish with ARL 67156, a non-hydrolyzable ATP analog and competitive Cd39 inhibitor (Fig. 3E) and used optogenetics to directly activate astroglia (fig. S7C). Consistent with our previous work (49), optogenetic stimulation of astroglia caused passivity in untreated fish, mimicking the effect of astroglial calcium increases during natural futility-induced passivity. However, pretreatment with ARL 67156 delayed passivity in response to optogenetic astroglial stimulation (Fig. 3G). While optogenetic stimulation of astroglia has been shown in some cases to elicit non-physiological calcium responses (58–60), the stimulation here elicits behavioral output similar to open-loop visual stimuli. These results indicate that the passivity-stimulating action of extracellular ATP is mediated by extracellular biochemical pathways that metabolize it into adenosine.

To directly visualize extracellular adenosine dynamics during futility-induced passivity, we generated a fish line expressing the extracellular green fluorescent adenosine sensor GRABAdo1.0 (32) in neurons and found that, as predicted by our behavioral experiments (Fig. 3E–G), extracellular adenosine rises during futile swims (Fig. 3H,I). Preventing astroglial activation, and therefore ATP release, by blocking α1-ARs with prazosin abolished futility-induced adenosine elevation (Fig. 3I, blue). Further, inhibiting metabolism of astroglial-secreted ATP into adenosine with ARL 67156, attenuated adenosine buildup during futility (Fig. 3I, red). Finally, inhibiting ATP-to-adenosine metabolism prevented GRABAdo1.0 elevation to exogenously applied ATP. (fig. S8A,B). These experiments indicate that adenosine elevation, downstream of astroglial ATP release, is a component of the futility-induced astroglial noradrenergic-to-purinergic pathway.

Adenosine acts as a signaling molecule in the central nervous system primarily by binding G-protein coupled adenosine receptors. To assess the involvement of adenosine receptors in the futility response, we performed pharmacological experiments to either drive or inhibit adenosine receptor signaling. We found that the adenosine receptor agonist 2-chloroadenosine increased passivity and decreased swim rate in both closed and open loop (fig. S8C–E), demonstrating that adenosine receptor activation is sufficient to trigger passivity. Non-specific inhibition of adenosine receptor signaling with caffeine, an adenosine receptor antagonist, suppressed open-loop passivity (Fig. 3J, left) but had no effect on open-loop struggle probability (Fig. 3J, right). The suppression of passivity did not result from effects upstream of astroglial calcium, as caffeine did not decrease astroglial calcium elevation nor astroglial ATP secretion in response to NE (if anything, both seemed to increase following caffeine treatment, but the effect was variable) (fig. S8F,G). Caffeine has off-target effects unrelated to its inhibition of adenosine receptors. However, we also found that directly buffering adenosine with the high-affinity sensor GRABAdo1.0 also resulted in a small but significant reduction in futility-induced passivity compared to wild-type siblings (fig. S8H, p = 0.0355). Finally, more specific blockade of A2B adenosine receptors, but not A1 receptors or A2A receptors, inhibited futility-induced passivity (Fig. 3K,L). Therefore, the astroglial noradrenergic-to-purinergic pathway recruited by futility implements the inhibitory, but not excitatory phase of the futility response primarily through A2 receptors on downstream neurons.

## Adenosine drives swim-suppressing neurons in the lateral medulla

**Figure 4.**
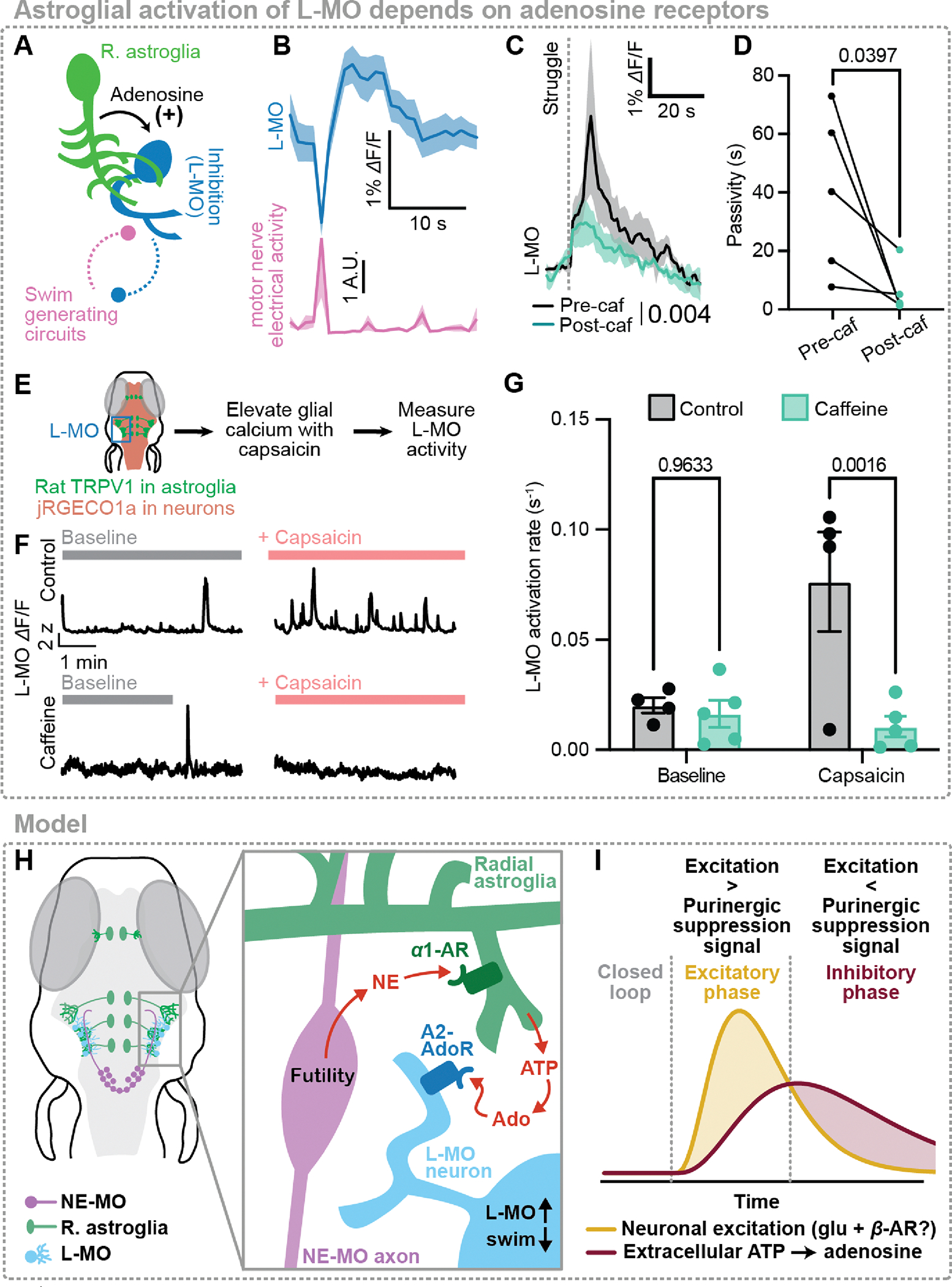
Adenosine persistently activates the swim-suppressing region L-MO. (**A**) Schematic: astroglial communication with L-MO, and mutual inhibition between L-MO and motor regions. (**B**) Example of L-MO neuronal activity anticorrelation to swim vigor in one fish. (**C**) Struggle-evoked L-MO activity before and after caffeine (100 μM), mean across five fish; N = 5; P = 0.004, Mann-Whitney on AUC from 0 – 60 s following struggle. (**D**) Mean of futility-triggered passivity durations before and after caffeine treatment. N = 5; P = 0.0397, paired t-test. (**E**) Schematic: activating astroglia in *Tg(gfap:TRPV1-eGFP;elavl3:JRGECO1a)* fish while imaging L-MO activity using light-sheet microscopy. (**F**) L-MO activity in 4 example fish treated with either vehicle control (top row) 100 *μ*M caffeine (an adenosine receptor blocker, bottom row) or vehicle control, either in baseline untreated condition (left) or with capsaicin (right). (**G**) Summary of rate of L-MO activation across all fish and conditions. N = 4 (control), 5 (caffeine); P = 0.9633 (baseline control vs. caffeine), 0.0016 (capsaicin control vs. caffeine), two-way ANOVA with Šídák’s multiple comparisons test. (**H**) Model: futility-triggered NE release drives astroglial ATP release. ATP is metabolized extracellularly into adenosine, and adenosine activates A2 adenosine receptors in L-MO to increase L-MO activity and suppress swimming. (**I**) Model: futility-related NE-MO firing drives fast excitation (yellow). NE mediates delayed inhibition (red) through astroglial activation, ATP release, and ATP-to-adenosine metabolism; eventually, inhibition overcomes fast excitation to drive the inhibitory phase of passivity. Thus, an astroglial noradrenergic-to-purinergic pathway mediates feedforward inhibition of the passivity response. All error bars and shaded error regions represent s.e.m.

Neurons are the cells that ultimately control swimming; therefore, we reasoned that futility-induced astroglial adenosine release acts on neural targets to suppress passivity. Because our pharmacological evidence supported a role for the A2B adenosine receptor (Fig. 3L), we performed in situ hybridization with probes targeting adora2b (mRNA for A2B adenosine receptor) transcripts and found, consistent with previous reports (61), adora2b expression in the midbrain and hindbrain, near the midline and in the subventricular zone (SVZ), as well as in bilaterally symmetrical hindbrain neuronal populations (fig. S9). Neuronal expression of adora2b appeared anatomically proximal to L-MO, a population that is activated by futility and suppresses swimming (Fig. 4A–B, fig. S10) (49). Imaging L-MO activity during behavior in Tg(elavl3:jRGECO1a) fish revealed that inhibition of adenosine receptors with caffeine reduced the magnitude and duration of persistent L-MO activation triggered by high-amplitude futile swims (Fig. 4C). This reduction was also reflected in a decrease in average futility-triggered passivity duration (Fig. 4D). Possible interpretations of the remaining struggle-induced L-MO activation is an incomplete block by caffeine or occurring via mechanisms independent of adenosine receptor activation. Furthermore, chemogenetic activation of astroglia using Tg(gfap:TRPV1-eGFP;elavl3:jRGECO1a) fish increased the rate of L-MO activation events, and the adenosine receptor antagonist caffeine inhibited this increase but had no effect on baseline L-MO activity (Fig. 4E–G). A caveat of this experiment is that our chemogenetic activation of astroglia causes astroglial calcium elevation across large areas of the brain; therefore, in this experiment we cannot rule out involvement of regions beyond L-MO in suppressing swimming. However, we previously demonstrated that L-MO is likely the primary driver of futility-induced passivity (49). Altogether, these data provide evidence that the purinergic signal released by astroglia exerts feedforward inhibition on motor circuits through the activation of inhibitory neurons in L-MO via A2B adenosine receptors (Fig. 4H,I).

## Discussion

Our work shows that the rapid state transition orchestrated by NE proceeds via astroglia through purinergic neuromodulation. We identify a functional logic connecting these two critical neuromodulatory systems. Noradrenergic neurons drive fast excitation but also recruit purinergic signaling, through astroglia, to implement delayed, feedforward inhibition. Thus, astroglia play a central role in coordinating across different neuromodulatory systems.

The NE-astroglia-purinergic pathway is recruited when actions become futile and a behavioral state change is necessary, analogous to NE-mediated transitions under the ‘global model failure’ conceptualization of NE function (18–20, 62). The components of this pathway – monoamine-triggered astroglial calcium signaling, astroglial ATP release, extracellular ATP-to-adenosine conversion, and adenosinergic suppression of neural activity – are ubiquitous across brain regions and species, which suggests that this conserved norepinephrine-astroglia-purinergic signaling motif (or NAP motif) could be a fundamental computational unit implementing feedforward inhibition over neuromodulatory timescales (63–65). Indeed, a companion paper by Lefton et al. (66), as well as several additional emerging lines of evidence (67, 68), demonstrates that NE’s well-known depressive effect on excitatory synapses in mice proceeds entirely through this same NE-astroglia-ATP/adenosine pathway. Our results, along with those of Lefton et al., argue that the neuromodulatory effects of NE, and perhaps other neuromodulators, must be reconsidered under the lens of astroglial modulation of neurophysiology and circuit dynamics.

Feedforward inhibition is widespread in synaptically coupled circuits (53, 54, 69, 70). Synaptic feedforward inhibition serves many functions, such as improving the spatial and temporal precision of neural coding (53, 54), gain control (69), and improving sensory acuity (70). Here, we show that feedforward inhibition can also be implemented through molecular circuits to play similar computational roles on much longer timescales (tens of seconds) that are complementary to the millisecond timescales of neuronal feedforward inhibition. Importantly, although astroglial activation by NE is widespread and quite synchronized, output diversification may occur at each step from ATP release, to ATP-to-adenosine metabolism, and finally to adenosine receptor activation. For example, adenosine receptor expression could be altered in different brain regions, the sensitivity of astroglia to norepinephrine could be modulated by local neuronal activity, or the activity of the extracellular enzymes that transform ATP into adenosine could be tuned, to modulate this extrasynaptic circuit motif in a context-dependent manner. Astroglial-mediated feedforward inhibition may therefore be similarly flexible and ubiquitous, but operate on slower timescales.

Over the past few decades, several lines of work have raised the possibility for an evolutionarily conserved role for the NAP motif in feedforward inhibition. Classical work has shown that an ATP-to-adenosine pathway acts in the spinal cord to inhibit locomotion in tadpoles (35), and more recent work suggests that astroglia contribute to spinal cord ATP release and locomotion suppression in rodents (71). Astroglia have also been shown to drive the inhibitory phases of a variety of episodic behaviors, such as sleep (72) and sensory-evoked arousal (30, 73). We speculate that the seemingly conserved role for astroglia in feedforward inhibition following rapid excitation may reflect – and may have arisen from – a fundamental astroglial function to regulate neuronal network excitability. Indeed, both astroglia and purinergic signaling play central roles in controlling excitability (64, 74), and astroglial and purinergic dysfunction is implicated in epileptic seizure generation (75). As astroglia possess multiple molecular pathways, such as those involved in potassium buffering, purinergic release, and glutamate metabolism (75, 76), to modulate neuronal excitability, an ancestral astroglial role in excitability regulation may have been appropriated to modulate circuit activity in many different behaviorally relevant contexts.

## Supplementary Material

Supplementary Materials

Movie S1

## Data Availability

Data and analysis code are available at Zenodo. https://doi.org/10.5281/zenodo.14278354
