# Supplementary material for: Norepinephrine changes behavioral state through astroglial purinergic signaling
Source: Science. Author manuscript; Available in PMC 2025 Jul 16. (PMC12265949; doi:10.1126/science.adq5233)
Supplement: Supplementary Materials [file NIHMS2086409-supplement-Supplementary_Materials.pdf]

Science
AAAS

# Supplementary Materials for

## Norepinephrine changes behavioral state through astroglial purinergic signaling

Alex B. Chen *et al*.

Corresponding authors: Alex B. Chen, abchen@g.harvard.edu; Misha B. Ahrens, ahrensm@janelia.hhmi.org

*Science* **388**, 769 (2025)
DOI: 10.1126/science.adq5233

**The PDF file includes:**

Materials and Methods
Figs. S1 to S10
Table S1
References

**Other Supplementary Material for this manuscript includes the following:**

MDAR Reproducibility Checklist
Movie S1

## Materials and Methods

Experimental model and subject details

Experiments were conducted in accordance with the guidelines of the National Institutes of Health. Animals were handled according IACUC protocols #1836 (Prober lab), #2729 (Engert lab), and 22-0216 (Ahrens lab). For all experiments in larval zebrafish, we used wild-type larval zebrafish (strains AB or WIK), aged 5–8 days post-fertilization (dpf), or transgenic fish (see Fish lines section). The sex of the fish is indeterminate at this age. Fish were raised in shallow Petri dishes, on a 14 h:10 h light:dark cycle at around 27°C, and fed ad libitum with paramecia after 4 dpf. All experiments were done during daylight hours (4–14 h after lights on). All protocols and procedures were approved by the Harvard University/Faculty of Arts and Sciences Standing Committee on the Use of Animals in Research and Teaching (Institutional Animal Care and Use Committee), and the Janelia Institutional Animal Care and Use Committee.

Fish lines

For all behavioral experiments we used:

Wild type - strains WIK and AB

For imaging of astroglial calcium we used:

Cytosolic, red calcium indicator. *Tg(gfap:jRGECO1a)* (*49*, *78*)

For imaging of neuronal calcium we used:

Nuclear-localized, green calcium indicator. *Tg(elavl3:H2B-jGCaMP7f)*$^{jf90}$ (*79*)

Cytosolic, red calcium indicator. *Tg(elavl3:jRGECO1a)* (*78*)

For optogenetic activation of norepinephrinergic neurons we used:

Channelrhodopsin expressed under dbh promoter. *Tg(dbh:KalTA4);Tg(UAS:CoChR-eGFP)* (*80*)

For chemogenetic activation of astroglia we used:

Rat transient receptor potential cation channel subfamily V member 1 (TRPV1) expressed under gfap promoter. *Tg(gfap:TRPV1-T2A-eGFP)*$^{jf64}$ (*50*)

For imaging of ATP we used:

GRAB$_{ATP1.0}$ (*57*) expressed under gfap promoter. *Tg(gfap:GRAB*$_{ATP1.0}$*)* (*this paper*)

For astroglial-specific inhibition of calcium signaling we used:

Human PMCA2 expressed under gfap promoter (*81*). *Tg(gfap:hPMCA2-mCherry)* (*this paper*)

For imaging of adenosine we used:

GRAB$_{Ado1.0}$ (*32*) expressed under elavl3 promoter. *Tg(elavl3:GRAB*$_{Ado1.0}$*)* (*this paper*)

For experiments in which multiple transgenes were required (e.g. simultaneous imaging of glial calcium and extracellular ATP), fish were crossed and double-positive offspring used.

Zebrafish transgenesis

We generated the *Tg(gfap:GRAB*$_{ATP1.0}$*)* and *Tg(elavl3:GRAB*$_{Ado1.0}$*)* lines used in this paper. The lines were generated in casper background (*82*) using the Tol2 method (*83*).

Embedding of larval zebrafish for tail-tracking experiments and confocal microscopy

Larval zebrafish aged 6-8 dpf were embedded in small round Petri dishes (e.g. Corning #351006) not treated for cell-culture use (for confocal imaging experiments). A solution of 2% low melting-point agarose (Sigma-Aldrich A9414) was prepared by heating agarose powder in near-boiling

filtered system water and agitating until fully dissolved. The 2% agarose solution fwas kept at 42-48 degrees Celsius. To embed fish, a small amount of 2% agarose solution was pipetted in the middle of a Petri dish. A larval zebrafish was then transferred using either a small glass or Pasteur pipette. Following the setting of the agar, the tail of the fish was freed by cutting away the agarose around the tail with a micro-scalpel (Fine Science Tools 10315-12).

Behavioral experiments with embedded larval zebrafish and visual stimulus delivery

For behavioral experiments using embedded larval zebrafish, we used a previously published, custom-build behavioral rig and custom-written code (*84*). Briefly, we illuminated the fish and its environment using infrared light-emitting diode panels (wavelength 940 nm, Cop Security). A video of the fish's tail was recorded using a camera (Grasshopper3-NIR, FLIR Systems) with a zoom lens (Zoom 7000, 18–108 mm, Navitar) and a long-pass filter (R72, Hoya). Tail position and swim bout kinematics were determined in real time by analyzing the position of ~25 equally spaced, user-defined key points along its length. Posture was determined and recorded in real-time at 90 Hz using custom-written Python scripts (Python 3.7, OpenCV 4.1). Swim bouts were detected in real time by calculating moving-window standard deviation of tail angle and thresholding. Detected swim bouts were used to deliver visual feedback via bottom-projection at 60 Hz (AAXA P300 Pico Projector).

Tracking of freely swimming larval zebrafish

For behavioral experiments with freely-swimming larval zebrafish, a previously published, custom-build behavioral rig and custom-written code (*84*) was used. The illumination and detection of freely swimming fish, as well as visual stimuli delivery, was the same as for behavioral experiments with embedded fish. To track the position of fish and determine swim bouts in real time at 90 Hz, we used custom-written Python scripts (Python 3.7, OpenCV 4.1) (*84*). The background of the camera image was subtracted and the body of the fish identified by center of mass. Orientation was determined as the axis of largest pixel variance in the identified body. Swim bouts were detected by computing a 50-ms rolling variance and identifying thresholded peaks.

Inclusion criteria for analyses

Fish were included for behavioral analyses if they appeared healthy and swam regularly during closed loop conditions in response to forward drifting grating (i.e. they performed the optomotor response similarly to previous publications (*45*, *46*)). Previously, Mu et al. reported that, under their experimental conditions, about 50 percent of fish did not perform futility-induced passivity and were therefore excluded from analyses (*49*). However, our conditions differed, particularly in that we did not paralyze fish. Under these conditions, almost all healthy fish (> 80%) exhibit futility-induced passivity, so performance of futility-induced passivity was not used as an inclusion criterion.

Passivity computation in zebrafish

As previously published, passive periods were operationally defined to be periods greater than 5 s in length in which the fish did not perform a swim bout (*49*). To obtain the open-loop passivity, the summed length of passive periods during the open-loop period of a trial was divided by open-loop period length, and the trial average calculated. For experiments in which multiple clutches of fish were used, we normalized treatment passivity to mean passivity of untreated controls.

Vigor computation and struggle analysis

For each detected swim, we computed swim vigor by calculating the mean vigor (rolling-window variance of tail angle) over the entire swim period. To compute average open-loop swim vigor, we averaged over all detected swims in the open-loop period. For panels involving multiple different experimental sessions, we normalized open-loop swim vigor by dividing by the mean open-loop vigor of the control group (Normalized swim vigor).

Struggles were defined to be swims characterized by particularly large vigor (>2.5 standard deviations). Struggle rate was computed to be the number of struggles divided by the length of open loop period to obtain open loop struggles per second.

Embedding of larval zebrafish for light-sheet microscopy experiments

Embedding protocol for light-sheet experiments was the same as described for tail-tracking and confocal microscopy experiments, except that the fish was immobilized in a custom-fabricated behavioral chamber compatible with our light-sheet microscope, as described previously (*49*). Agarose was removed around the head of the fish to allow for light-sheet penetration, and a small amount of agarose was removed over the dorsal part of the fish's tail to enable access for suction electrodes used for fictive behavioral recording.

Pharmacological treatment of larval zebrafish

Larval zebrafish (6-8 dpf) were transferred to Petri dishes containing either vehicle or the experimental pharmacological compound and incubated for at least 1 hour before being embedded for behavioral or imaging experiments. Information regarding dose, source, and references of pharmacological compounds used can be found in Table S1.

Pharmacological treatment in anesthetized larval zebrafish

Larval zebrafish (6-8 dpf) were immersed in external solution (prepared as described in (*85*)) containing 160µg/mL of MS-222 and adjusted to a pH of 7.4. After embedding the fish in 2% agarose, a small amount of agarose was removed on the dorsal side of the head. Using a fire polished injection pipette, a small incision was made to expose the brain to the external solution. Different compounds were then applied to the external solution above the fish using a 10uL pipette.

Freely swimming optogenetic/uncaging experiments

For experiments involving optogenetic perturbations in freely swimming fish, as well as NPE-ATP uncaging experiments, fish were transferred to a water droplet on the lid of a Petri dish under an upright widefield fluorescence microscope (Olympus MVX10) and fish position imaged with an integrated CMOS camera (IDS Imaging UI-3370CP-NIR). Optogenetic activation and uncaging was performed using an LED lamp (X-Cite 120 LED mini) and appropriate chromatic filter.

HCR *in situ* hybridization staining

HCR- FISH staining was done as described previously in a recent study (*86*). Briefly, AB WT *mitfa-/-* larvae were euthanized with 0.2% MS-222, then fixed in 4% paraformaldehyde in Dulbecco's phosphate-buffered saline (DPBS) overnight at 4°C with gentle shaking. To end the fixing process, the larvae were washed three times with DPBST (1X DPBS + 0.1% Tween 20), each session for 5 minutes. They were then dehydrated and made permeable by placing them in ice-cold 100% methanol at -20 °C for 10 minutes. Following this, the samples were washed first

with a 50% methanol/DPBST mix and then with a 25% methanol/DPBST mix, each for 5 minutes, and finally rehydrated with five 5-minute washes in DPBST. For prehybridization, 5 larvae were placed in a 2-ml Eppendorf tube with a prewarmed hybridization buffer for 30 minutes at 37 °C. This buffer was then replaced with the 2pmol *adora2b* HCR probe set (Molecular Instruments, Inc., *adora2b* sequence: NCBI BC163683) in hybridization buffer and incubated for 12 hours at 37 °C with gentle shaking. Afterward, the larvae were washed four times with a probe wash buffer at 37 °C and twice with 5X SSCT(5X sodium chloride sodium citrate + 0.1% Tween 20) at room temperature. Pre-amplification involved incubating the sample in 500 µl amplification buffer at room temperature for 30 minutes. Then, 30 pmol of B2 488 HCR amplifiers (h1 and h2 hairpins, Molecular Instruments, Inc.) were prepared by incubating 10 µl of 3 µM stock hairpins in 95°C for 90 s and cooling them down to room temperature for 30 mins in a dark environment. The hairpin solution was prepared by transferring B2 488 h1 and B2 488 h2 hairpins to 500 µl of amplification buffer.The samples were incubated in this solution for 12 hours in the dark at room temperature. The next day, excess hairpins were washed off with three 20-minute washes in 5X SSCT at room temperature. All reagents were bought from Molecular Instruments. The animals were mounted in 1.5% agarose and imaged with a 3i spinning disk confocal microscope (spinning head: Yokogawa CSU-W1, camera: Hamamatsu Orca Flash 4.0 V3) using a 20x objective. The signal was imaged using a 488 nm laser and the signal was collected and imaged using a [510-540] filter set. The images were processed using Fiji.

Statistics

Statistical analyses used in main figure panels are stated in figure legends. For low sample size, exact tests were used. Sample sizes were determined using power analyses based on effect sizes observed in previous literature. Analyses used in supplementary figures are described in supplementary figure legends. In all figures, error bars and shaded error regions represent s.e.m.

**Fig. S1.**

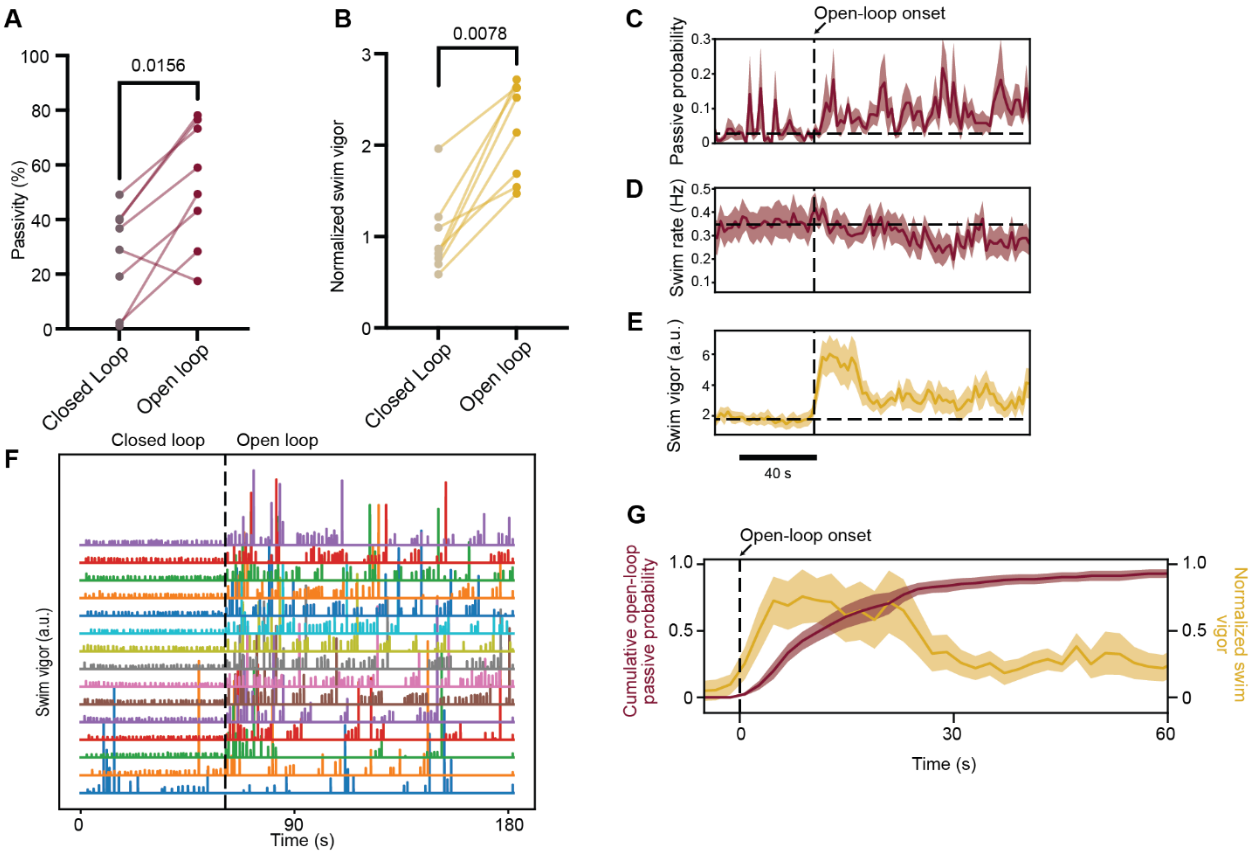

A
100
80
60
40
20
0
Passivity (%)
0.0156
Closed Loop
Open loop
B
3
2
1
0
Normalized swim vigor
0.0078
Closed Loop
Open loop
C
Open-loop onset
Passive probability
0.3
0.2
0.1
0
D
Swim rate (Hz)
0.5
0.4
0.3
0.2
0.1
E
Swim vigor (a.u.)
6
4
2
40 s
F
Closed loop
Open loop
Swim vigor (a.u.)
0
90
180
Time (s)
G
Open-loop onset
Cumulative open-loop passive probability
1.0
0.5
0
Normalized swim vigor
0
30
60
Time (s)


170

**Figure S1. Additional characterization of swimming behavior in closed- and open-loop.** (**A**) Percentage of either closed or open loop spent passive (> 5 seconds without swimming). Wilcoxon signed-rank test. (**B**) Swim vigor/intensity in closed and open loop of the same fish in (**A**), normalized to the closed loop mean. Wilcoxon signed-rank test. (**C-E**) Open-loop-triggered passive probability (**C**), swim rate (**D**), and swim vigor (**E**). Dashed vertical line denotes transition from closed to open loop. 8 fish, 10 trials per fish. (**F**) Example closed- and open-loop swimming behavior for an individual fish. Each row corresponds to one trial; dashed vertical line denotes transition from closed to open loop. Note the variable onset of open loop from trial to trial. (**G**) Cumulative open-loop probability of passivity onset (red) as well as normalized swim vigor (yellow) in the first 60 seconds of open loop. Cumulative open-loop probability was calculated by determining the earliest time after open-loop onset in each trial when a fish first became passive, then averaging onset across trials and fish. Traces represent mean across same fish as (**A**) and (**B**), and shaded error regions represent s.e.m.Type or paste caption here. Create a page break and paste in the figure above the caption.

**Fig. S2.**

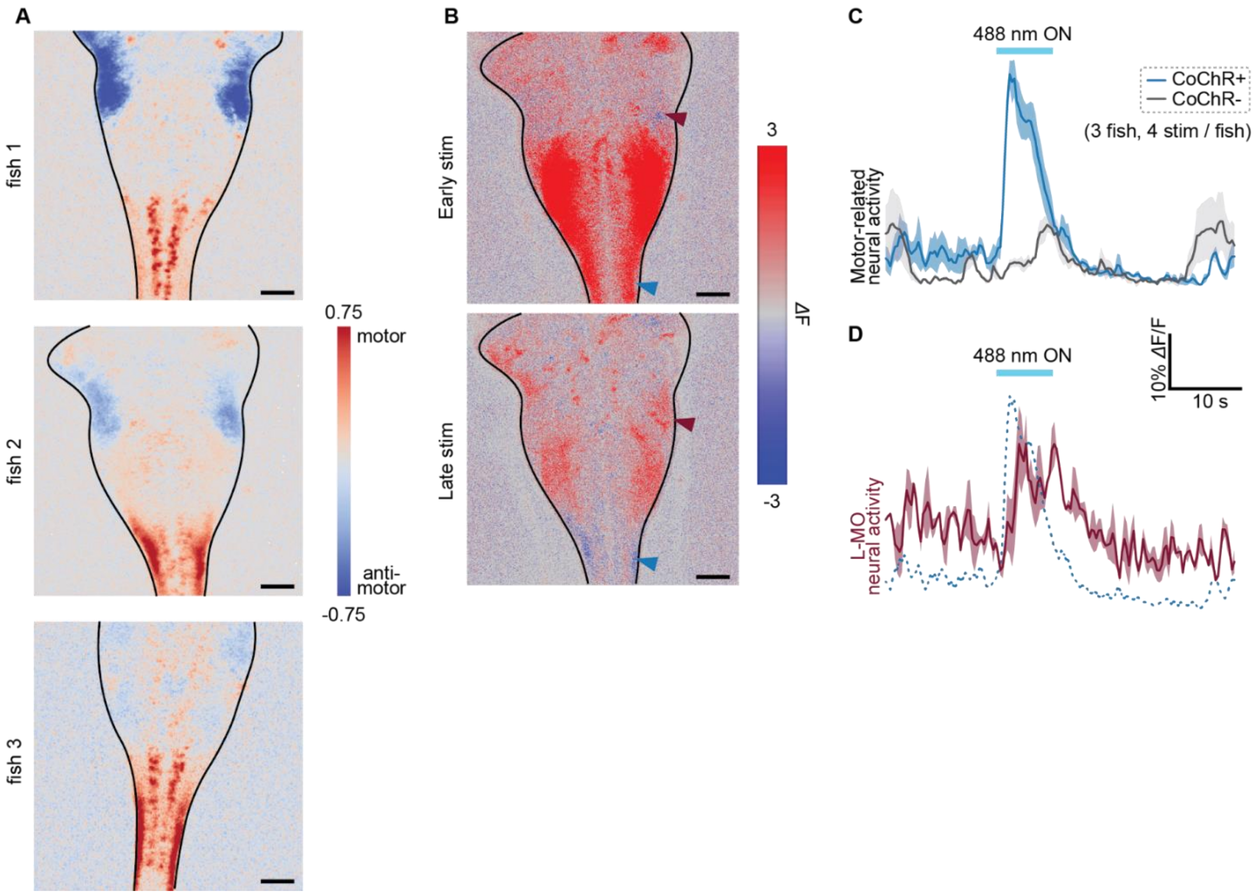
A
fish 1
fish 2
fish 3
0.75
motor
anti-
motor
-0.75
B
Early stim
Late stim
3
ΔF
-3
C
488 nm ON
CoChR+
CoChR-
(3 fish, 4 stim / fish)
Motor-related
neural activity
D
488 nm ON
10% ΔF/F
10 s
L-MO
neural activity

**Figure S2**. **Optogenetic stimulation of noradrenergic neurons.** (**A**) Putatively motor- and anti-motor-correlated pixels in confocal micrographs of three fish. Scale bar denotes 50 $\mu$m. (**B**) Single fish, single trial example of change in fluorescence in the hindbrain in response to optogenetic stimulation of noradrenergic neurons using *Tg(dbh:CoChR-eGFP)* fish, relative to pre-stimulation baseline. Top image shows average response in the ~1 s immediately following 488 nm light ON. Bottom image shows response average response in the ~2 s following light offset. Maroon arrowheads denote L-MO, blue arrowheads denote a motor-related area. Scale bar denotes 50 $\mu$m. (**C**) Motor-related activity triggered on 488 nm laser onset across all fish and trials for CoChR positive and negative fish (3 fish of each genotype, 4 trials per fish). (**D**) Motor-anticorrelated activity triggered on 488 nm laser onset across all fish and trials for CoChR positive fish (red trace) overlaid with mean motor-related activity shown in panel (C). All error bars and shaded error regions represent s.e.m.

**Fig. S3.**

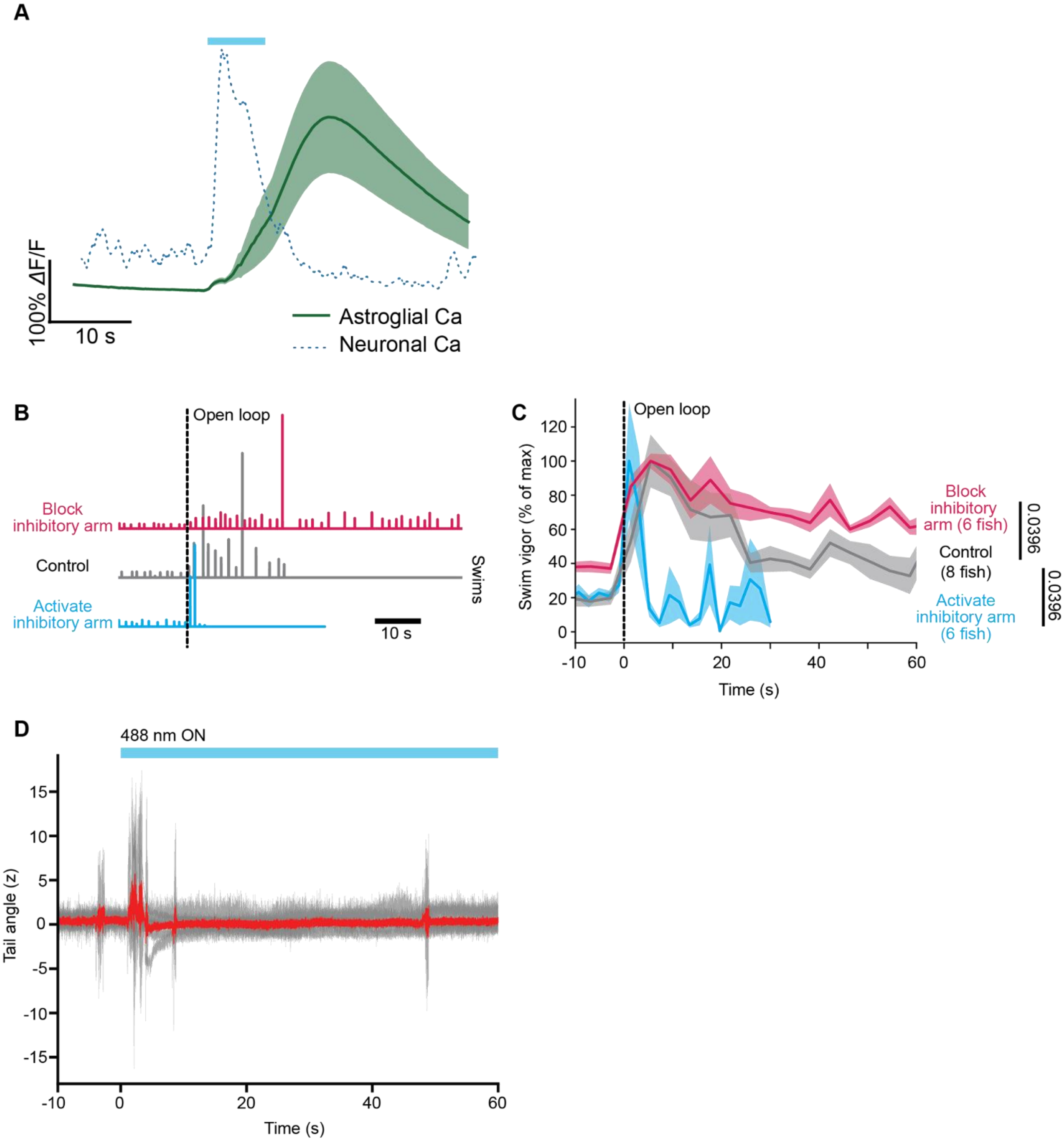
A
100% ΔF/F
10 s
Astroglial Ca
Neuronal Ca
B
Open loop
Block
inhibitory arm
Control
Activate
inhibitory arm
Swims
10 s
C
Open loop
Swim vigor (% of max)
120
100
80
60
40
20
0
-10
0
20
40
60
Time (s)
Block
inhibitory
arm (6 fish)
Control
(8 fish)
Activate
inhibitory arm
(6 fish)
0.0396
0.0396
D
488 nm ON
Tail angle (z)
15
10
5
0
-5
-10
-15
-10
0
20
40
60
Time (s)

**Figure S3. Comparison of optogenetically-evoked astroglial and neuronal calcium events and effect of manipulating inhibitory arm on window of excitation.** (**A**) Mean and s.e.m. of optogenetically evoked neuronal $Ca^{2+}$ (3 fish, 4 stims per fish) is plotted. Blue bar indicates period of time of 488 nm light on. Dotted blue line represents mean optogenetically evoked neuronal $Ca^{2+}$ of motor regions, reproduced from fig. S2C. (**B**) Representative trials in a control fish, a fish in which the inhibitory arm (astroglial calcium signaling) is blocked with 100 μM prazosin (red trace), and a fish in which the inhibitory arm is activated optogenetically at open-loop onset (blue trace). (**C**) Average across fish and trials in which the inhibitory arm (astroglial calcium signaling) is blocked with 100 μM prazosin (red trace), and a fish in which the inhibitory arm is activated optogenetically at open-loop onset (blue trace). Prazosin causes a slight increase in closed-loop swim vigor, reflected in an upward shift before time = 0. Statistical test: Kruskal-Wallis test on duration of vigor increase (full-width-half-max of vigor increase). (**D**) Z-scored tail amplitude for 6 fish (gray traces) and average (red trace). At time = 0, a constant 488 nm LED is used to optogenetically activate noradrenergic neurons (fish line Tg(dbh:KalTA4; UAS:CoChR-eGFP)); the LED remains on for 60 seconds (blue bar).

**Fig. S4.**

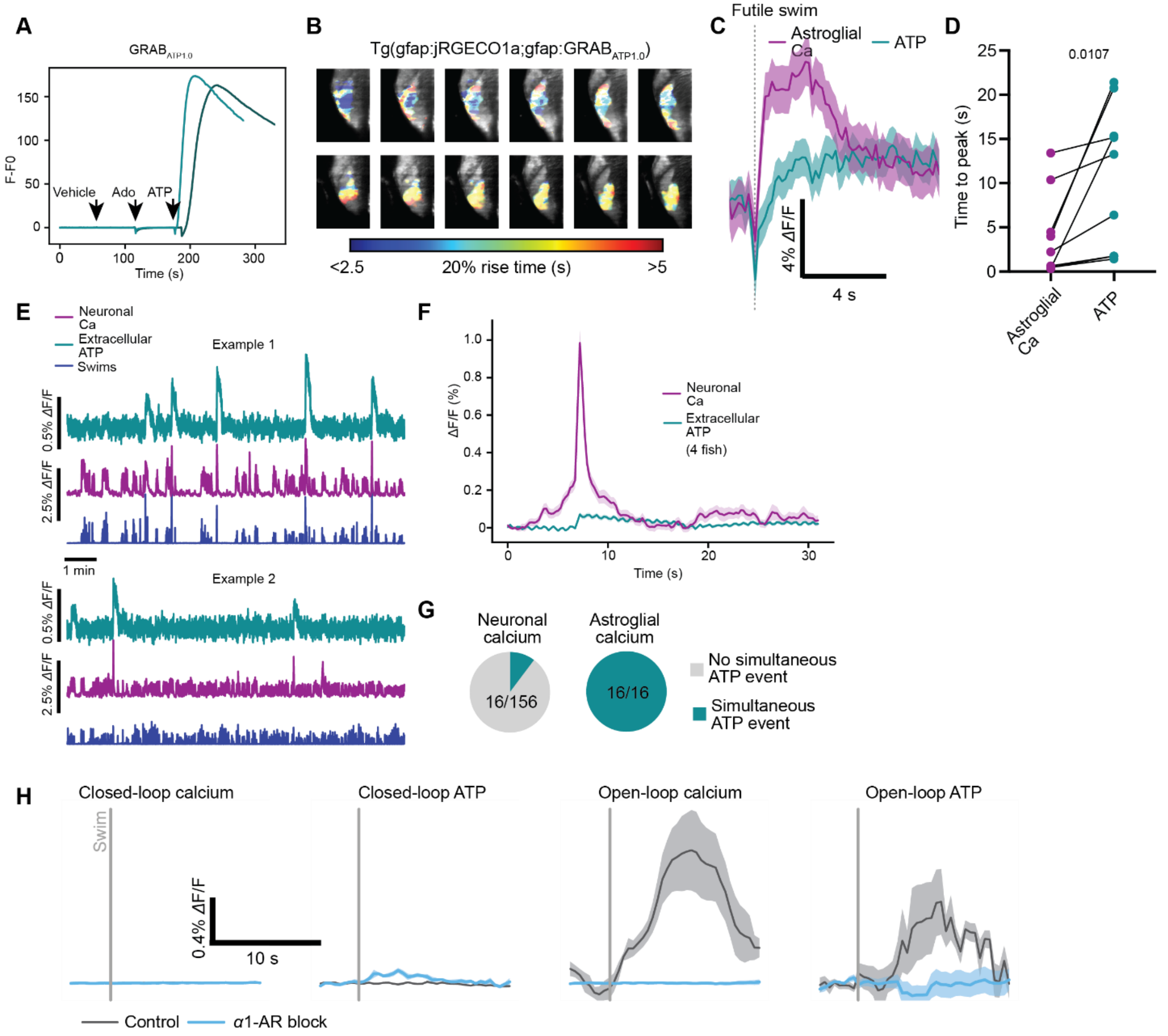

A
GRAB$_{ATP1.0}$
F-F0
Vehicle
Ado
ATP
Time (s)
B
Tg(gfap:jRGECO1a;gfap:GRAB$_{ATP1.0}$)
<2.5
20% rise time (s)
>5
C
Futile swim
Astroglial Ca
ATP
4% ΔF/F
4 s
D
0.0107
Time to peak (s)
Astroglial Ca
ATP
E
Neuronal Ca
Extracellular ATP
Swims
Example 1
0.5% ΔF/F
2.5% ΔF/F
1 min
Example 2
F
ΔF/F (%)
Neuronal Ca
Extracellular ATP
(4 fish)
Time (s)
G
Neuronal calcium
Astroglial calcium
16/156
16/16
No simultaneous ATP event
Simultaneous ATP event
H
Closed-loop calcium
Closed-loop ATP
Open-loop calcium
Open-loop ATP
Swim
0.4% ΔF/F
10 s
Control
α1-AR block

**Figure S4. Additional evidence that astroglia, not neurons, release ATP in response to NE.** (**A**) Baseline subtracted fluorescence traces for 2 Tg(gfap:GRABATP1.0) fish after addition of either vehicle (DMSO 0.1%), adenosine (100µM) or ATP (100µM) under anesthetized conditions (blue and orange represent the two individual fish; black arrows represent approximate timing, precise timing can be determined by brief downward deflection upon addition of ATP/Ado). Note the lack of responses to vehicle or adenosine, but a strong response to ATP. (**B**) Characterization of 20% rise time of intracellular astroglial $Ca^{2+}$ signal (top) and extracellular ATP signal around astroglia (bottom) in simultaneously imaged *Tg(gfap:jRGECO1a;gfap:GRAB$_{ATP1.0}$)* fish. (**C**) Futile swim-triggered extracellular ATP and astroglial $Ca^{2+}$ signals collected simultaneously. (**D**) Average time-to-peak of ATP and $Ca^{2+}$ signals in the same fish shown in (B). Wilcoxon rank sum test. (**E**) Example fluorescence signals from GRABATP1.0 (green), neuronal jRGECO1 signal (red) and swims (blue) simultaneously recorded from an example fish in open loop during two periods of the experiment. (**F**) Average of neuronal calcium and extracellular signals across 4 fish, triggered on detected neuronal calcium peaks. Shaded error bars represent s.e.m. (**G**) Proportion of neuronal or astroglial calcium events associated with a concurrent extracellular ATP elevation. Only 16/156 neuronal firing events were accompanied by extracellular ATP elevation, while 16/16 astroglial calcium events were accompanied by extracellular ATP elevation (N = 5 fish for neuronal Ca, 5 fish for astroglial Ca). (**H**) Swim-triggered fluorescence traces of GRAB$_{ATP1.0}$ (extracellular ATP) and jRGECO1 (intracellular astroglial calcium) signal in closed- and open-loop measured in Tg(gfap:jRGECO1a; gfap:GRABATP1.0) fish treated with either 100 µM prazosin (blue) or vehicle control (gray). Mean jRGECO1 signal in neurons before and after puffing of methoxamine, an $\alpha$1-AR agonist, with 167 mg/L MS-222, a sodium channel blocker, in the bath.

**Fig. S5.**

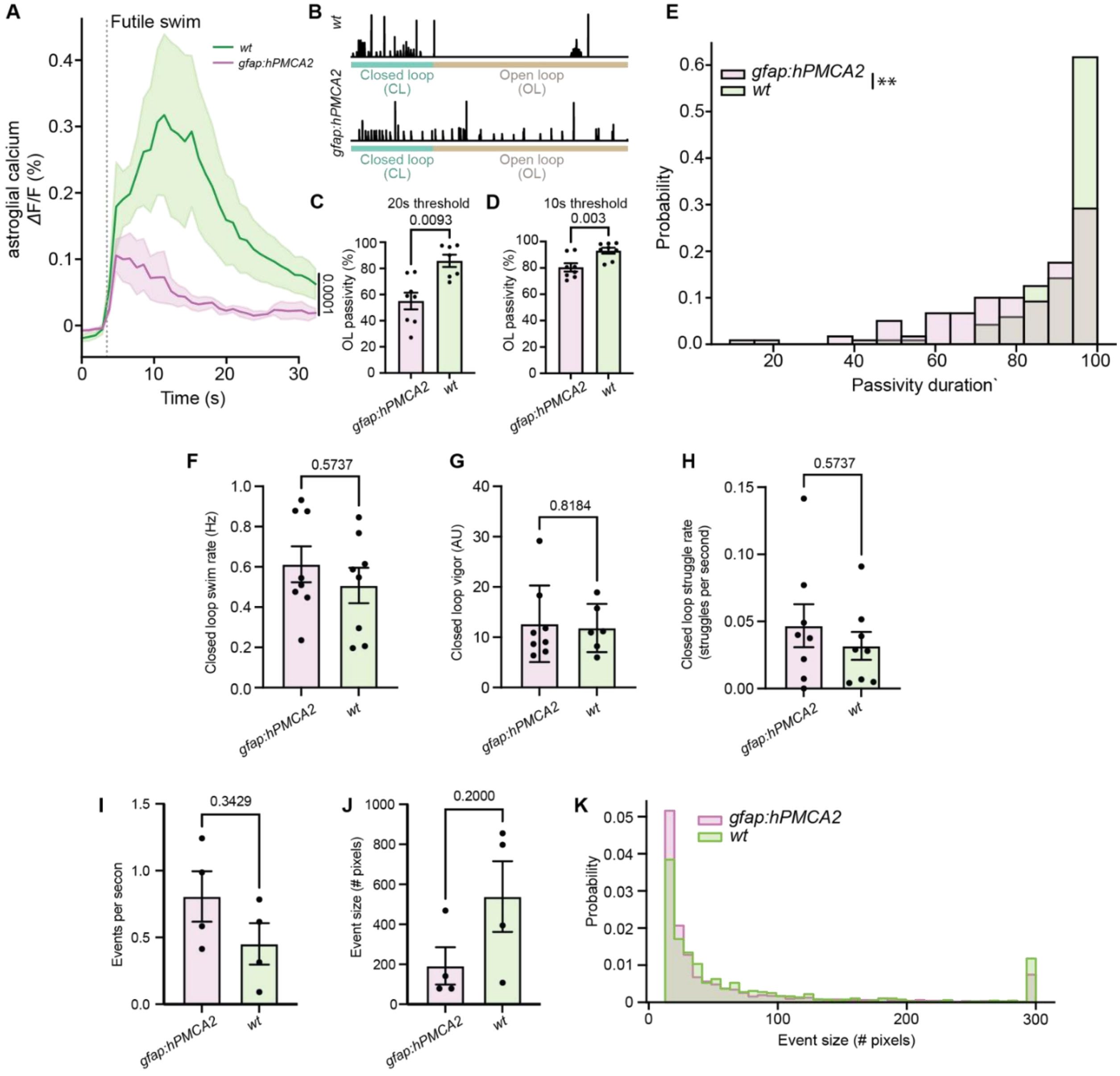
A
Futile swim
wt
gfap:hPMCA2
astroglial calcium
ΔF/F (%)
0.0001
Time (s)
B
wt
Closed loop
(CL)
Open loop
(OL)
gfap:hPMCA2
Closed loop
(CL)
Open loop
(OL)
C
20s threshold
0.0093
OL passivity (%)
gfap:hPMCA2
wt
D
10s threshold
0.003
OL passivity (%)
gfap:hPMCA2
wt
E
gfap:hPMCA2
wt
**
Probability
Passivity duration`
F
0.5737
Closed loop swim rate (Hz)
gfap:hPMCA2
wt
G
0.8184
Closed loop vigor (AU)
gfap:hPMCA2
wt
H
0.5737
Closed loop struggle rate
(struggles per second)
gfap:hPMCA2
wt
I
0.3429
Events per secon
gfap:hPMCA2
wt
J
0.2000
Event size (# pixels)
gfap:hPMCA2
wt
K
gfap:hPMCA2
wt
Probability
Event size (# pixels)

**Figure S5. Effect of hPMCA on astroglial calcium, behavior, animal health, and baseline astroglial activity.** (**A**) Futile swim-triggered astroglial calcium signal in sibling controls expressing only GCaMP in astroglia (*wt*) or fish expressing the calcium extruder hPMCA2 specifically in astroglia (*Tg(gfap:hPMCA2-mCherry)*). p = 0.0001, Mann-Whitney on AUC of 30 s following futile swim, n = 8 fish, each genotype. (**B**) Swim traces in open and closed loop for example *wt* and *Tg(gfap:hPMCA2-mCherry)* fish. (**C-D**) Proportion open loop period spent passive for *wt* and *Tg(gfap:hPMCA2-mCherry)* fish, using (**C**) a 20 second or (**D**) a 10 second no swim cutoff to score passivity. Mann-Whitney. (**E**) Histograms showing passivity duration distribution for *wt* and *Tg(gfap:hPMCA2-mCherry)* fish from panels **B-D**. ** $p < 0.01$, Kolmogorov–Smirnov test. (**F-H**) Closed-loop swim rate (**F**), vigor (**G**) or struggle rates (**H**) for controls and Tg(gfap:hPMCA2). (**I-K**) Frequency (**I**) and size (**J,K**) of spontaneous glial calcium events (B,C) in control and *Tg(gfap:hPMCA2)* fish. F-J: Mann-Whitney.

**Fig. S6.**

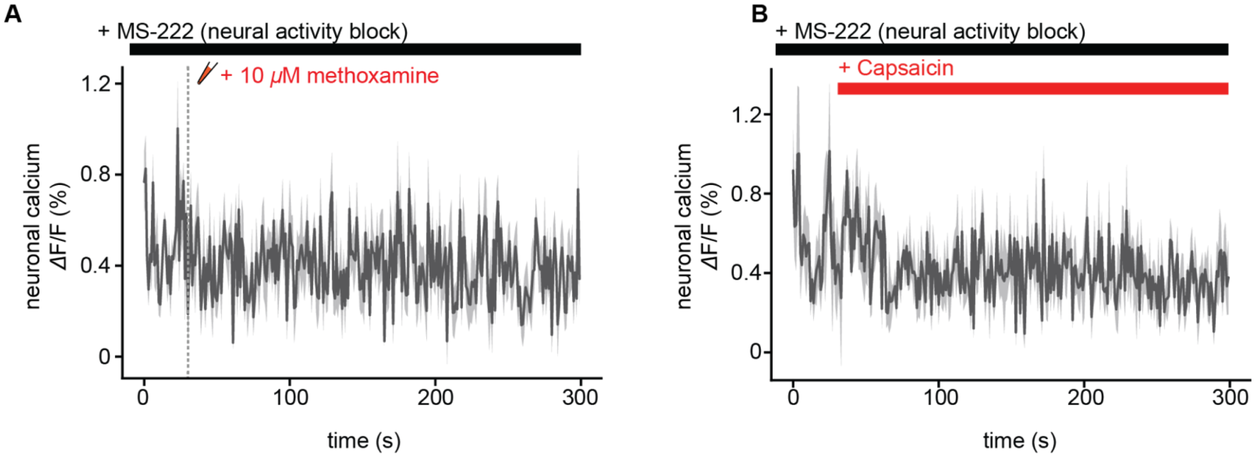
A
+ MS-222 (neural activity block)
+ 10 μM methoxamine
neuronal calcium
ΔF/F (%)
1.2
0.8
0.4
0
0
100
200
300
time (s)
B
+ MS-222 (neural activity block)
+ Capsaicin
neuronal calcium
ΔF/F (%)
1.2
0.8
0.4
0
0
100
200
300
time (s)

**Figure S6. Activating astroglia has no effect on neural activity in anesthetized fish.**
(**A**) Mean jRGECO1 signal in neurons of five fish before and after puffing of methoxamine, an $\alpha$1-AR agonist, with 167 mg/L MS-222, a sodium channel blocker, in the bath. (**B**) Mean jRGECO1 signal in neurons of five fish before and after application of capsaicin in *Tg(gfap:TRPV1-eGFP;elavl3:jRGECO1)* fish, with 167 mg/L MS-222, a sodium channel blocker, in the bath. All shaded error regions and error bars denote s.e.m.

**Fig. S7.**

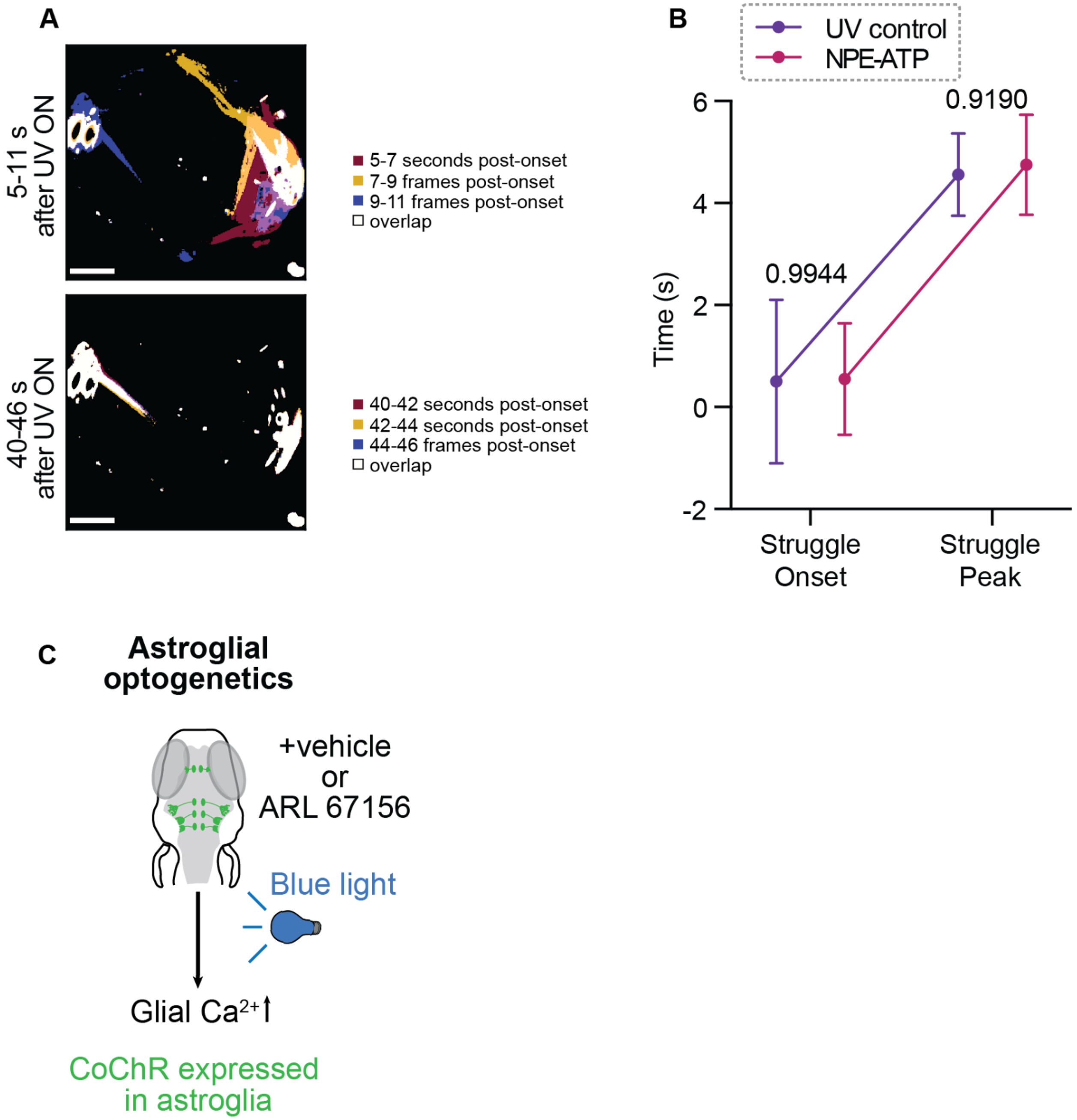
A
5-11 s after UV ON
40-46 s after UV ON
5-7 seconds post-onset
7-9 frames post-onset
9-11 frames post-onset
overlap
40-42 seconds post-onset
42-44 seconds post-onset
44-46 frames post-onset
overlap
B
UV control
NPE-ATP
Time (s)
6
4
2
0
-2
0.9944
0.9190
Struggle Onset
Struggle Peak
C
Astroglial optogenetics
+vehicle or ARL 67156
Blue light
Glial Ca2+
CoChR expressed in astroglia

**Figure S7. Additional analyses of NPE-ATP experiments.**

(**A**) Example maximum projections of an untreated fish during the same experiments described in Fig. 3A. Video frames taken either during the early stimulation period (top, 11 - 20 frames, or 5.5 – 10 seconds post UV onset) or late stimulation period (bottom, 80 - 89 frames or 40 - 44.5 seconds post UV onset) are color-coded by time and projected. White areas denote overlap, or objects that show little motion over time. Smaller white fragments are reflection artifacts. Scale bar 5 mm. (**B**) Quantification of struggle onset following UV exposure and time to peak swim speed following UV exposure for control fish or fish treated with NPE-ATP. Two-way ANOVA with Sidak's multiple comparisons test. (**C**) Experimental schematic: astroglia optogenetically activated in freely swimming *Tg(gfap:CoChR-eGFP)* fish treated with 1 mM ARL 67156 or vehicle. All error bars and shaded error regions represent s.e.m.

290 **Fig. S8.**

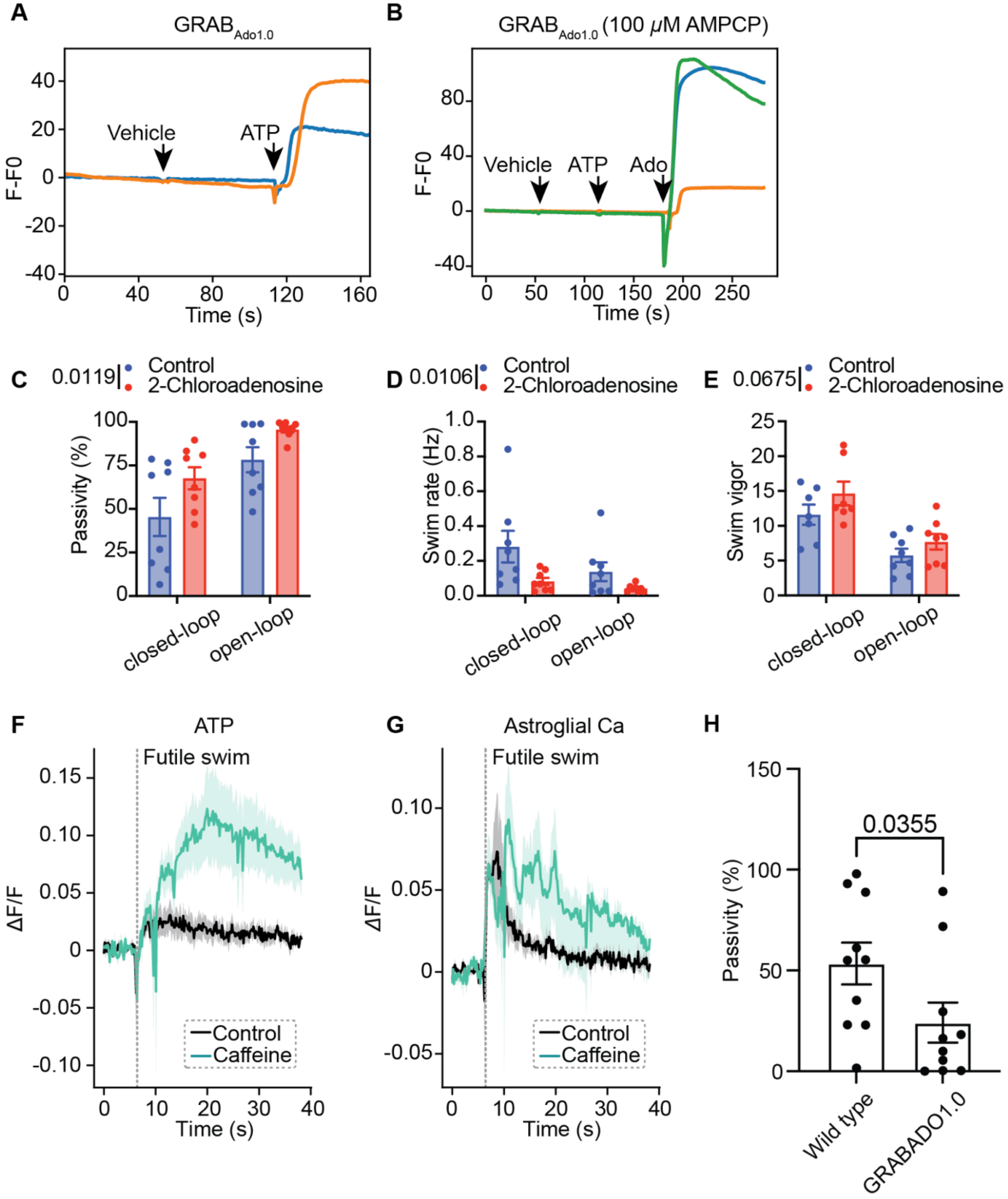
A
GRAB Ado1.0
F-F0
Vehicle
ATP
Time (s)
B
GRAB Ado1.0 (100 μM AMPCP)
Vehicle
ATP
Ado
C
0.0119
Control
2-Chloroadenosine
Passivity (%)
closed-loop
open-loop
D
0.0106
Swim rate (Hz)
E
0.0675
Swim vigor
F
ATP
Futile swim
ΔF/F
Control
Caffeine
G
Astroglial Ca
H
0.0355
Wild type
GRABADO1.0

**Figure S8. Further evidence that secreted ATP drives passivity via extracellular conversion into adenosine.** (**A**) Responses to either vehicle or ATP in two Tg(elavl3:GRABADO) fish, with no response to vehicle and a delayed response to ATP, presumably through extracellular conversion of ATP to adenosine. (**B**) Three Tg(elavl3:GRABADO) fish were treated with a Cd39 blocker, AMPCP (100μM) to block extracellular conversion of ATP into adenosine. Under these conditions, there was no response to either vehicle or ATP, but a strong response to adenosine. (**C-E**) Effect of 1 mM 2-chloroadenosine on (**C**) Passivity, **(D)** swim rate, and (**E**) swim vigor in both closed and open loop. Two-way ANOVA. (**F-G**) Futile swim-triggered extracellular ATP elevation (**F**) or astroglial calcium elevation (**G**) in fish treated with either vehicle or 100 $\mu$M caffeine. N = 4 control, 5 caffeine-treated. All error bars and shaded error regions represent s.e.m. (**H**) Open-loop passivity in *Tg(elavl3:GRABADO$_{1.0}$)* fish and wildtype sibling controls. Statistical test: Mann-Whitney.

**Fig. S9**

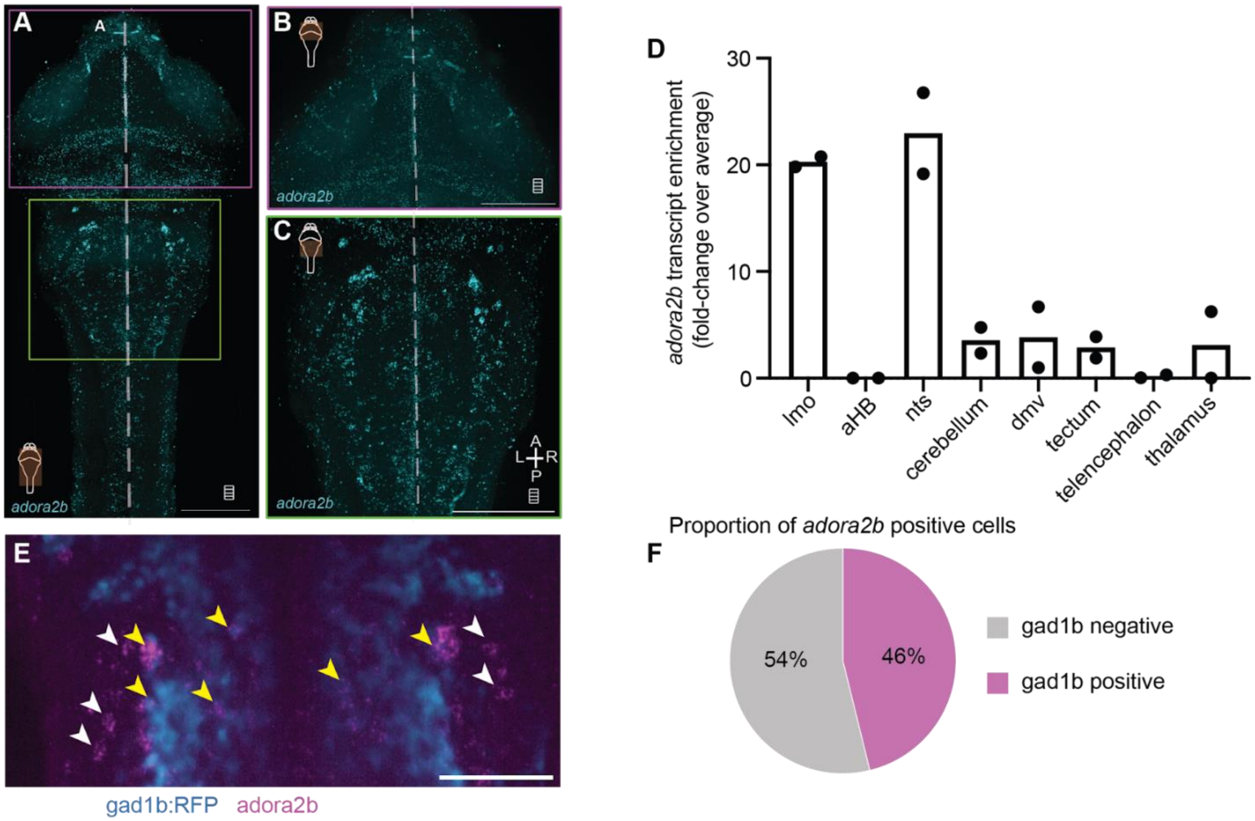

A
A
adora2b
B
adora2b
C
adora2b
A
L
R
P
D
adora2b transcript enrichment (fold-change over average)
30
20
10
0
lmo
aHB
nts
cerebellum
dmv
tectum
telencephalon
thalamus
E
gad1b:RFP
adora2b
Proportion of adora2b positive cells
F
54%
46%
gad1b negative
gad1b positive

**Figure S9. HCR in situ staining of adora2b expression in larval zebrafish showing expression in the hindbrain.** (**A**) A typical substack maximum Z projection of adora2b expression in larval zebrafish brain and rostral spinal cord. Scale bar = 100 µm, maximum Z projection of 170 planes of 0.5 µm step size). (**B,C**) Zoomed confocal micrographs of adora2b expression in the (**B**) midbrain and in the (**C**) hindbrain of larval zebrafish corresponding to the same colored boxes in (A). Scale bar = 100 µm, maximum Z projection of 171 planes of 0.5 µm step size for (B) and 110 planes of 0.5 µm step size for (C). A - anterior, P - posterior, L - left, R - right, max Z projections are visualized by a rectangle with multiple lines and midline is represented with vertical dashed lines. All error bars and shaded error regions represent s.e.m. (**D**) *adora2b* expression levels (normalized to region volume) in 8 example regions in 2 fish, plotted as fold-change over average expression level across the entire brain. (**E**) Crop of single plane in L-MO region in a Tg(gad1b:LoxP-RFP-LoxP-GFP) fish (cyan) following HCR in situ probing for adora2b expression (magenta). Arrowheads show adora2b positive cells that are gad1b negative (white) or gad1b positive (yellow). (**F**) Proportion of adora2b positive cells that were either gad1b negative or gad1b positive across two fish.

325 **Fig. S10**

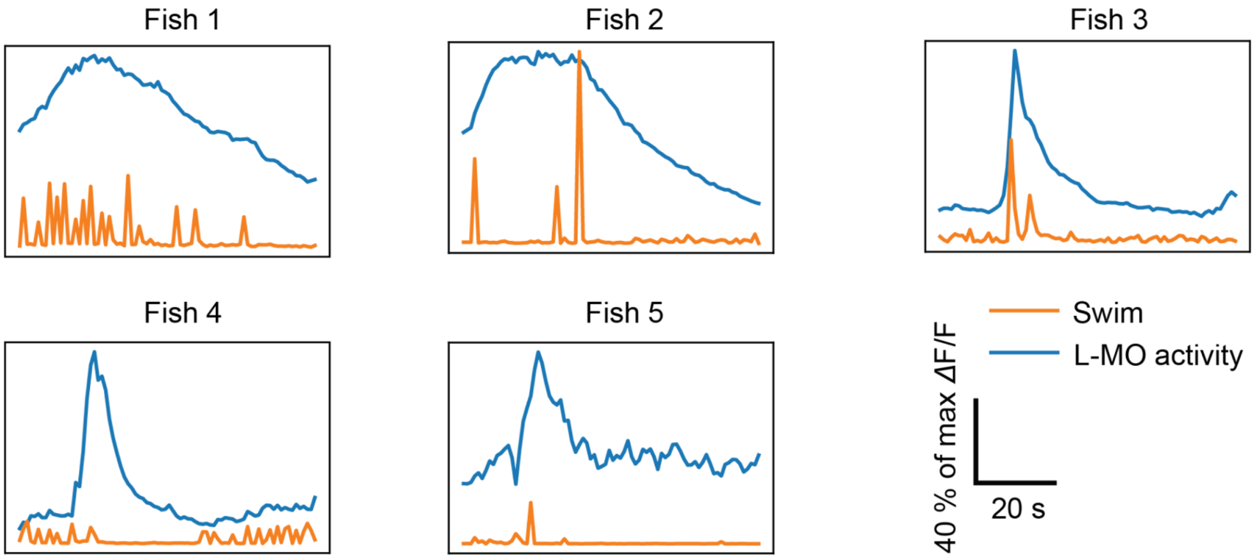
Fish 1
Fish 2
Fish 3
Fish 4
Fish 5
Swim
L-MO activity
40 % of max ΔF/F
20 s

**Figure S10. Average L-MO peak-triggered L-MO activity (blue) and swims (orange) in 5 example fish.** High L-MO activity is associated with decreased swimming.

**Table S1.**

| **Compound** | **Vehicle** | **Stock [ ]** | **Final [ ]** | **Reference** | **Source** |
|---|---|---|---|---|---|
| **MS-222 (Tricaine)** | Water | 160 mg/mL | 160μg/mL | (*87*) | Sigma E10521 |
| **Prazosin HCl** | Water | 1 mM | 100 μM | (*49*) | Sigma P7791 |
| **Propranolol HCl** | Water | 1mM | 100 μM | (*88*) | Sigma P0884 |
| **Methoxamine HCl** | DMSO | 1mM | 10 μM | (*49*) | Sigma M6524 |
| **Capsaicin** | DMSO | 200μM | 200nM | (*49*) | Sigma M2028 |
| **NPEC-ATP** | Water | 100mM | 100 μM | * | Thermo A1048 |
| **Suramin HCl** | Water | 100mM | 100 μM | (*89*) | Sigma S2671 |
| **AMPCP** | Water | 100mM | 100 μM | * | Sigma M3763 |
| **ARL-67156** | Water | 100mM | 100 μM | * | Sigma A265 |
| **Caffeine** | Water | 100mM | 100 μM | (*90*) | Sigma C0750 |
| **DPCPX** | DMSO | 100mM | 100 μM | *in adults (*91*) | Sigma C101 |
| **MRS-1754** | DMSO | 100mM | 100 μM | (*92*) | Sigma M6316 |
| **2-Chloroadenosine** | Water | - | 1mM | (*33*) | Sigma C5134 |
| **SCH-58261** | DMSO | 100mM | 100 μM | (*92*) | Sigma S4568 |

**Table S1.** List, sources, and concentrations of compounds used in pharmacological experiments. * denotes no record of testing in larval zebrafish.

**Movie S1.**

An example trial and fish swimming in closed and open loop, demonstrating tail tracking. Closed and open loop periods are labeled as are periods of normal swimming in closed loop, as well as the excitatory and inhibitory phases of the open loop response, as defined in Figure 1. Overlaid trace is tail angle.

**References and notes**


1. M. V. Bennett, Y. Nakajima, G. D. Pappas, Physiology and ultrastructure of electrotonic junctions. I. Supramedullary neurons. *J. Neurophysiol.* **30**, 161–179 (1967). doi:10.1152/jn.1967.30.2.161 Medline

2. A. Bhattacharya, U. Aghayeva, E. G. Berghoff, O. Hobert, Plasticity of the electrical connectome of *C. elegans*. *Cell* **176**, 1174–1189.e16 (2019). doi:10.1016/j.cell.2018.12.024 Medline

3. S. J. Smith, U. Sümbül, L. T. Graybuck, F. Collman, S. Seshamani, R. Gala, O. Gliko, L. Elabbady, J. A. Miller, T. E. Bakken, J. Rossier, Z. Yao, E. Lein, H. Zeng, B. Tasic, M. Hawrylycz, Single-cell transcriptomic evidence for dense intracortical neuropeptide networks. *eLife* **8**, e47889 (2019). doi:10.7554/eLife.47889 Medline

4. M. Lovett-Barron, A. S. Andalman, W. E. Allen, S. Vesuna, I. Kauvar, V. M. Burns, K. Deisseroth, Ancestral circuits for the coordinated modulation of brain state. *Cell* **171**, 1411–1423.e17 (2017). doi:10.1016/j.cell.2017.10.021 Medline

5. J. C. Marques, M. Li, D. Schaak, D. N. Robson, J. M. Li, Internal state dynamics shape brainwide activity and foraging behaviour. *Nature* **577**, 239–243 (2020). doi:10.1038/s41586-019-1858-z Medline

6. C. I. Bargmann, E. Marder, From the connectome to brain function. *Nat. Methods* **10**, 483–490 (2013). doi:10.1038/nmeth.2451 Medline

7. F. Randi, A. K. Sharma, S. Dvali, A. M. Leifer, Neural signal propagation atlas of *Caenorhabditis elegans*. *Nature* **623**, 406–414 (2023). doi:10.1038/s41586-023-06683-4 Medline

8. L. Ripoll-Sánchez, J. Watteyne, H. Sun, R. Fernandez, S. R. Taylor, A. Weinreb, B. L. Bentley, M. Hammarlund, D. M. Miller 3rd, O. Hobert, I. Beets, P. E. Vértes, W. R. Schafer, The neuropeptidergic connectome of *C. elegans*. *Neuron* **111**, 3570–3589.e5 (2023). doi:10.1016/j.neuron.2023.09.043 Medline

9. S. L. Hooper, E. Marder, Modulation of a central pattern generator by two neuropeptides, proctolin and FMRFamide. *Brain Res.* **305**, 186–191 (1984). doi:10.1016/0006-8993(84)91138-7 Medline

10. S. R. Yeh, R. A. Fricke, D. H. Edwards, The effect of social experience on serotonergic modulation of the escape circuit of crayfish. *Science* **271**, 366–369 (1996). doi:10.1126/science.271.5247.366 Medline

11. P. S. Katz, P. A. Getting, W. N. Frost, Dynamic neuromodulation of synaptic strength intrinsic to a central pattern generator circuit. *Nature* **367**, 729–731 (1994). doi:10.1038/367729a0 Medline

12. J. S. Coggan, T. M. Bartol, E. Esquenazi, J. R. Stiles, S. Lamont, M. E. Martone, D. K. Berg, M. H. Ellisman, T. J. Sejnowski, Evidence for ectopic neurotransmission at a neuronal synapse. *Science* **309**, 446–451 (2005). doi:10.1126/science.1108239 Medline

13. G. Mountoufaris, A. Nair, B. Yang, D.-W. Kim, A. Vinograd, S. Kim, S. W. Linderman, D. J. Anderson, A line attractor encoding a persistent internal state requires neuropeptide

signaling. *Cell* **187**, 5998–6015.e18 (2024). doi:10.1016/j.cell.2024.08.015 Medline

14. J. Nagai, X. Yu, T. Papouin, E. Cheong, M. R. Freeman, K. R. Monk, M. H. Hastings, P. G. Haydon, D. Rowitch, S. Shaham, B. S. Khakh, Behaviorally consequential astrocytic regulation of neural circuits. *Neuron* **109**, 576–596 (2021). doi:10.1016/j.neuron.2020.12.008 Medline

15. E. A. Bushong, M. E. Martone, Y. Z. Jones, M. H. Ellisman, Protoplasmic astrocytes in CA1 stratum radiatum occupy separate anatomical domains. *J. Neurosci.* **22**, 183–192 (2002). doi:10.1523/JNEUROSCI.22-01-00183.2002 Medline

16. G. Perea, M. Navarrete, A. Araque, Tripartite synapses: Astrocytes process and control synaptic information. *Trends Neurosci.* **32**, 421–431 (2009). doi:10.1016/j.tins.2009.05.001 Medline

17. U. S. V. Euler, A sympathomometic pressor substance in animal organ extracts. *Nature* **156**, 18–19 (1945). doi:10.1038/156018b0

18. R. Jordan, The locus coeruleus as a global model failure system. *Trends Neurosci.* **47**, 92–105 (2024). doi:10.1016/j.tins.2023.11.006 Medline

19. R. Jordan, G. B. Keller, The locus coeruleus broadcasts prediction errors across the cortex to promote sensorimotor plasticity. *eLife* **12**, RP85111 (2023). doi:10.7554/eLife.85111 Medline

20. D. G. R. Tervo, M. Proskurin, M. Manakov, M. Kabra, A. Vollmer, K. Branson, A. Y. Karpova, Behavioral variability through stochastic choice and its gating by anterior cingulate cortex. *Cell* **159**, 21–32 (2014). doi:10.1016/j.cell.2014.08.037 Medline

21. S. J. Sara, S. Bouret, Orienting and reorienting: The locus coeruleus mediates cognition through arousal. *Neuron* **76**, 130–141 (2012). doi:10.1016/j.neuron.2012.09.011 Medline

22. G. Aston-Jones, J. D. Cohen, An integrative theory of locus coeruleus-norepinephrine function: Adaptive gain and optimal performance. *Annu. Rev. Neurosci.* **28**, 403–450 (2005). doi:10.1146/annurev.neuro.28.061604.135709 Medline

23. M. E. Hasselmo, C. Linster, M. Patil, D. Ma, M. Cekic, Noradrenergic suppression of synaptic transmission may influence cortical signal-to-noise ratio. *J. Neurophysiol.* **77**, 3326–3339 (1997). doi:10.1152/jn.1997.77.6.3326 Medline

24. V. Zerbi, A. Floriou-Servou, M. Markicevic, Y. Vermeiren, O. Sturman, M. Privitera, L. von Ziegler, K. D. Ferrari, B. Weber, P. P. De Deyn, N. Wenderoth, J. Bohacek, Rapid reconfiguration of the functional connectome after chemogenetic locus coeruleus activation. *Neuron* **103**, 702–718.e5 (2019). doi:10.1016/j.neuron.2019.05.034 Medline

25. E. Bülbring, J. H. Burn, An action of adrenaline on transmission in sympathetic ganglia, which may play a part in shock. *J. Physiol.* **101**, 289–303 (1942). doi:10.1113/jphysiol.1942.sp003983 Medline

26. A. Uribe-Arias, R. Rozenblat, E. Vinepinsky, E. Marachlian, A. Kulkarni, D. Zada, M. Privat, D. Topsakalian, S. Charpy, V. Candat, S. Nourin, L. Appelbaum, G. Sumbre, Radial astrocyte synchronization modulates the visual system during behavioral-state transitions. *Neuron* **111**, 4040–4057.e6 (2023). doi:10.1016/j.neuron.2023.09.022 Medline

27. L. K. Bekar, W. He, M. Nedergaard, Locus coeruleus alpha-adrenergic-mediated activation of cortical astrocytes in vivo. *Cereb. Cortex* **18**, 2789–2795 (2008). doi:10.1093/cercor/bhn040 Medline

28. F. Ding, J. O'Donnell, A. S. Thrane, D. Zeppenfeld, H. Kang, L. Xie, F. Wang, M. Nedergaard, α1-Adrenergic receptors mediate coordinated Ca2+ signaling of cortical astrocytes in awake, behaving mice. *Cell Calcium* **54**, 387–394 (2013). doi:10.1016/j.ceca.2013.09.001 Medline

29. Z. Ma, T. Stork, D. E. Bergles, M. R. Freeman, Neuromodulators signal through astrocytes to alter neural circuit activity and behaviour. *Nature* **539**, 428–432 (2016). doi:10.1038/nature20145 Medline

30. M. E. Reitman, V. Tse, X. Mi, D. D. Willoughby, A. Peinado, A. Aivazidis, B.-E. Myagmar, P. C. Simpson, O. A. Bayraktar, G. Yu, K. E. Poskanzer, Norepinephrine links astrocytic activity to regulation of cortical state. *Nat. Neurosci.* **26**, 579–593 (2023). doi:10.1038/s41593-023-01284-w Medline

31. T. Porkka-Heiskanen, R. E. Strecker, M. Thakkar, A. A. Bjorkum, R. W. Greene, R. W. McCarley, Adenosine: A mediator of the sleep-inducing effects of prolonged wakefulness. *Science* **276**, 1265–1268 (1997). doi:10.1126/science.276.5316.1265 Medline

32. W. Peng, Z. Wu, K. Song, S. Zhang, Y. Li, M. Xu, Regulation of sleep homeostasis mediator adenosine by basal forebrain glutamatergic neurons. *Science* **369**, eabb0556 (2020). doi:10.1126/science.abb0556 Medline

33. A. Suppermpool, D. G. Lyons, E. Broom, J. Rihel, Sleep pressure modulates single-neuron synapse number in zebrafish. *Nature* **629**, 639–645 (2024). doi:10.1038/s41586-024-07367-3 Medline

34. O. Pascual, K. B. Casper, C. Kubera, J. Zhang, R. Revilla-Sanchez, J.-Y. Sul, H. Takano, S. J. Moss, K. McCarthy, P. G. Haydon, Astrocytic purinergic signaling coordinates synaptic networks. *Science* **310**, 113–116 (2005). doi:10.1126/science.1116916 Medline

35. N. Dale, D. Gilday, Regulation of rhythmic movements by purinergic neurotransmitters in frog embryos. *Nature* **383**, 259–263 (1996). doi:10.1038/383259a0 Medline

36. M. Wall, N. Dale, Activity-dependent release of adenosine: A critical re-evaluation of mechanism. *Curr. Neuropharmacol.* **6**, 329–337 (2008). doi:10.2174/157015908787386087 Medline

37. D. van Calker, K. Biber, K. Domschke, T. Serchov, The role of adenosine receptors in mood and anxiety disorders. *J. Neurochem.* **151**, 11–27 (2019). doi:10.1111/jnc.14841 Medline

38. L. Weltha, J. Reemmer, D. Boison, The role of adenosine in epilepsy. *Brain Res. Bull.* **151**, 46–54 (2019). doi:10.1016/j.brainresbull.2018.11.008 Medline

39. T. V. Dunwiddie, L. Diao, W. R. Proctor, Adenine nucleotides undergo rapid, quantitative conversion to adenosine in the extracellular space in rat hippocampus. *J. Neurosci.* **17**, 7673–7682 (1997). doi:10.1523/JNEUROSCI.17-20-07673.1997 Medline

40. R. de Ceglia, A. Ledonne, D. G. Litvin, B. L. Lind, G. Carriero, E. C. Latagliata, E. Bindocci, M. A. Di Castro, I. Savtchouk, I. Vitali, A. Ranjak, M. Congiu, T. Canonica,

W. Wisden, K. Harris, M. Mameli, N. Mercuri, L. Telley, A. Volterra, Specialized astrocytes mediate glutamatergic gliotransmission in the CNS. *Nature* **622**, 120–129 (2023). doi:10.1038/s41586-023-06502-w Medline

41. D. Lovatt, Q. Xu, W. Liu, T. Takano, N. A. Smith, J. Schnermann, K. Tieu, M. Nedergaard, Neuronal adenosine release, and not astrocytic ATP release, mediates feedback inhibition of excitatory activity. *Proc. Natl. Acad. Sci. U.S.A.* **109**, 6265–6270 (2012). doi:10.1073/pnas.1120997109 Medline
42. L. Yang, Y. Qi, Y. Yang, Astrocytes control food intake by inhibiting AGRP neuron activity via adenosine A1 receptors. *Cell Rep.* **11**, 798–807 (2015). doi:10.1016/j.celrep.2015.04.002 Medline
43. M. J. Broadhead, G. B. Miles, Bi-directional communication between neurons and astrocytes modulates spinal motor circuits. *Front. Cell. Neurosci.* **14**, 30 (2020). doi:10.3389/fncel.2020.00030 Medline
44. G. R. J. Gordon, D. V. Baimoukhametova, S. A. Hewitt, W. R. A. K. J. S. Rajapaksha, T. E. Fisher, J. S. Bains, Norepinephrine triggers release of glial ATP to increase postsynaptic efficacy. *Nat. Neurosci.* **8**, 1078–1086 (2005). doi:10.1038/nn1498 Medline
45. M. B. Orger, M. C. Smear, S. M. Anstis, H. Baier, Perception of Fourier and non-Fourier motion by larval zebrafish. *Nat. Neurosci.* **3**, 1128–1133 (2000). doi:10.1038/80649 Medline
46. E. Yang, M. F. Zwart, B. James, M. Rubinov, Z. Wei, S. Narayan, N. Vladimirov, B. D. Mensh, J. E. Fitzgerald, M. B. Ahrens, A brainstem integrator for self-location memory and positional homeostasis in zebrafish. *Cell* **185**, 5011–5027.e20 (2022). doi:10.1016/j.cell.2022.11.022 Medline
47. N. Jurisch-Yaksi, E. Yaksi, C. Kizil, Radial glia in the zebrafish brain: Functional, structural, and physiological comparison with the mammalian glia. *Glia* **68**, 2451–2470 (2020). doi:10.1002/glia.23849 Medline
48. J. Chen, K. E. Poskanzer, M. R. Freeman, K. R. Monk, Live-imaging of astrocyte morphogenesis and function in zebrafish neural circuits. *Nat. Neurosci.* **23**, 1297–1306 (2020). doi:10.1038/s41593-020-0703-x Medline
49. Y. Mu, D. V. Bennett, M. Rubinov, S. Narayan, C.-T. Yang, M. Tanimoto, B. D. Mensh, L. L. Looger, M. B. Ahrens, Glia accumulate evidence that actions are futile and suppress unsuccessful behavior. *Cell* **178**, 27–43.e19 (2019). doi:10.1016/j.cell.2019.05.050 Medline
50. M. Duque, A. B. Chen, S. Narayan, D. E. Olson, M. C. Fishman, F. Engert, M. B. Ahrens, Astroglial mediation of fast-acting antidepressant effect in zebrafish. bioRxiv 52099 [Preprint] (2022). https://doi.org/10.1101/2022.12.29.522099.
51. L. Rinaman, Hindbrain noradrenergic A2 neurons: Diverse roles in autonomic, endocrine, cognitive, and behavioral functions. *Am. J. Physiol. Regul. Integr. Comp. Physiol.* **300**, R222–R235 (2011). doi:10.1152/ajpregu.00556.2010 Medline
52. G. Moruzzi, H. W. Magoun, Brain stem reticular formation and activation of the EEG. *Electroencephalogr. Clin. Neurophysiol.* **1**, 455–473 (1949). doi:10.1016/0013-

4694(49)90219-9 Medline

53. F. Pouille, M. Scanziani, Enforcement of temporal fidelity in pyramidal cells by somatic feed-forward inhibition. *Science* **293**, 1159–1163 (2001). doi:10.1126/science.1060342 Medline

54. W. Mittmann, U. Koch, M. Häusser, Feed-forward inhibition shapes the spike output of cerebellar Purkinje cells. *J. Physiol.* **563**, 369–378 (2005). doi:10.1113/jphysiol.2004.075028 Medline

55. A. V. Gourine, V. Kasymov, N. Marina, F. Tang, M. F. Figueiredo, S. Lane, A. G. Teschemacher, K. M. Spyer, K. Deisseroth, S. Kasparov, Astrocytes control breathing through pH-dependent release of ATP. *Science* **329**, 571–575 (2010). doi:10.1126/science.1190721 Medline

56. T. A. Babola, S. Li, Z. Wang, C. J. Kersbergen, A. B. Elgoyhen, T. M. Coate, D. E. Bergles, Purinergic signaling controls spontaneous activity in the auditory system throughout early development. *J. Neurosci.* **41**, 594–612 (2021). doi:10.1523/JNEUROSCI.2178-20.2020 Medline

57. Z. Wu, K. He, Y. Chen, H. Li, S. Pan, B. Li, T. Liu, F. Xi, F. Deng, H. Wang, J. Du, M. Jing, Y. Li, A sensitive GRAB sensor for detecting extracellular ATP in vitro and in vivo. *Neuron* **110**, 770–782.e5 (2022). doi:10.1016/j.neuron.2021.11.027 Medline

58. W.-H. Cho, E. Barcelon, S. J. Lee, Optogenetic glia manipulation: Possibilities and future prospects. *Exp. Neurobiol.* **25**, 197–204 (2016). doi:10.5607/en.2016.25.5.197 Medline

59. E. Gerasimov, A. Erofeev, A. Borodinova, A. Bolshakova, P. Balaban, I. Bezprozvanny, O. L. Vlasova, Optogenetic activation of astrocytes-effects on neuronal network function. *Int. J. Mol. Sci.* **22**, 9613 (2021). doi:10.3390/ijms22179613 Medline

60. S. A. Sloan, B. A. Barres, Looks can be deceiving: Reconsidering the evidence for gliotransmission. *Neuron* **84**, 1112–1115 (2014). doi:10.1016/j.neuron.2014.12.003 Medline

61. W. Boehmler, J. Petko, M. Woll, C. Frey, B. Thisse, C. Thisse, V. A. Canfield, R. Levenson, Identification of zebrafish A2 adenosine receptors and expression in developing embryos. *Gene Expr. Patterns* **9**, 144–151 (2009). doi:10.1016/j.gep.2008.11.006 Medline

62. C. Li, T. Sun, Y. Zhang, Y. Gao, Z. Sun, W. Li, H. Cheng, Y. Gu, N. Abumaria, A neural circuit for regulating a behavioral switch in response to prolonged uncontrollability in mice. *Neuron* **111**, 2727–2741.e7 (2023). doi:10.1016/j.neuron.2023.05.023 Medline

63. M. Corkrum, A. Covelo, J. Lines, L. Bellocchio, M. Pisansky, K. Loke, R. Quintana, P. E. Rothwell, R. Lujan, G. Marsicano, E. D. Martin, M. J. Thomas, P. Kofuji, A. Araque, Dopamine-evoked synaptic regulation in the nucleus accumbens requires astrocyte activity. *Neuron* **105**, 1036–1047.e5 (2020). doi:10.1016/j.neuron.2019.12.026 Medline

64. S. Pittolo, S. Yokoyama, D. D. Willoughby, C. R. Taylor, M. E. Reitman, V. Tse, Z. Wu, R. Etchenique, Y. Li, K. E. Poskanzer, Dopamine activates astrocytes in prefrontal cortex via α1-adrenergic receptors. *Cell Rep.* **40**, 111426 (2022). doi:10.1016/j.celrep.2022.111426 Medline

65. T. Deemyad, J. Lüthi, N. Spruston, Astrocytes integrate and drive action potential firing in

inhibitory subnetworks. *Nat. Commun.* **9**, 4336 (2018). doi:10.1038/s41467-018-06338-3 Medline

66. K. B. Lefton, Y. Wu, A. Yen, T. Okuda, Y. Zhang, Y. Dai, S. Walsh, R. Manno, J. D. Dougherty, V. K. Samineni, J. Dougherty, P. C. Simpson, T. Papouin, Norepinephrine signals through astrocytes to modulate synapses. *Science* **388**, 776 (2025). doi:10.1126/scienceadq5480

67. Q. Xin, J. Wang, J. Zheng, Y. Tan, X. Jia, Z. Ni, J. Feng, Z. Wu, Y. Li, X. Li, H. Ma, H. Hu, Neuron-astrocyte coupling in lateral habenula mediates depressive-like behaviors. *Cell* 10.1016/j.cell.2025.04.010 (2025). doi:10.1016/j.cell.2025.04.010 Medline

68. G. T. Drummond, A. Natesan, M. Celotto, J. Shih, P. Ojha, Y. Osako, J. Park, G. O. Sipe, K. R. Jenks, V. Breton-Provencher, P. C. Simpson, S. Panzeri, M. Sur, Cortical norepinephrine-astrocyte signaling critically mediates learned behavior. bioRxiv 620009 [Preprint] (2024); https://doi.org/10.1101/2024.10.24.620009.

69. S. R. Olsen, R. I. Wilson, Lateral presynaptic inhibition mediates gain control in an olfactory circuit. *Nature* **452**, 956–960 (2008). doi:10.1038/nature06864 Medline

70. M. Wehr, A. M. Zador, Synaptic mechanisms of forward suppression in rat auditory cortex. *Neuron* **47**, 437–445 (2005). doi:10.1016/j.neuron.2005.06.009 Medline

71. D. Acton, G. B. Miles, Stimulation of glia reveals modulation of mammalian spinal motor networks by adenosine. *PLOS ONE* **10**, e0134488 (2015). doi:10.1371/journal.pone.0134488 Medline

72. M. M. Halassa, C. Florian, T. Fellin, J. R. Munoz, S.-Y. Lee, T. Abel, P. G. Haydon, M. G. Frank, Astrocytic modulation of sleep homeostasis and cognitive consequences of sleep loss. *Neuron* **61**, 213–219 (2009). doi:10.1016/j.neuron.2008.11.024 Medline

73. J. Lines, E. D. Martin, P. Kofuji, J. Aguilar, A. Araque, Astrocytes modulate sensory-evoked neuronal network activity. *Nat. Commun.* **11**, 3689 (2020). doi:10.1038/s41467-020-17536-3 Medline

74. A. Badimon, H. J. Strasburger, P. Ayata, X. Chen, A. Nair, A. Ikegami, P. Hwang, A. T. Chan, S. M. Graves, J. O. Uweru, C. Ledderose, M. G. Kutlu, M. A. Wheeler, A. Kahan, M. Ishikawa, Y.-C. Wang, Y. E. Loh, J. X. Jiang, D. J. Surmeier, S. C. Robson, W. G. Junger, R. Sebra, E. S. Calipari, P. J. Kenny, U. B. Eyo, M. Colonna, F. J. Quintana, H. Wake, V. Gradinaru, A. Schaefer, Negative feedback control of neuronal activity by microglia. *Nature* **586**, 417–423 (2020). doi:10.1038/s41586-020-2777-8 Medline

75. C. Diaz Verdugo, S. Myren-Svelstad, E. Aydin, E. Van Hoeymissen, C. Deneubourg, S. Vanderhaeghe, J. Vancraeynest, R. Pelgrims, M. I. Cosacak, A. Muto, C. Kizil, K. Kawakami, N. Jurisch-Yaksi, E. Yaksi, Glia-neuron interactions underlie state transitions to generalized seizures. *Nat. Commun.* **10**, 3830 (2019). doi:10.1038/s41467-019-11739-z Medline

76. T. Miyashita, K. Murakami, E. Kikuchi, K. Ofusa, K. Mikami, K. Endo, T. Miyaji, S. Moriyama, K. Konno, H. Muratani, Y. Moriyama, M. Watanabe, J. Horiuchi, M. Saitoe, Glia transmit negative valence information during aversive learning in *Drosophila*. *Science* **382**, eadf7429 (2023). doi:10.1126/science.adf7429 Medline

77. Data and analysis code for: A. B. Chen, M. Duque, A. Rymbek, M. Dhanasekar, V. M. Wang, X. Mi, L. Tocquer, S. Narayan, E. Marquez Legorreta, M. Eddison, G. Yu, C. Wyart, D. Prober, F. Engert, M. B. Ahrens, Norepinephrine changes behavioral state through astroglial purinergic signaling, Zenodo (2025); https://doi.org/10.5281/zenodo.14278354.

78. H. Dana, B. Mohar, Y. Sun, S. Narayan, A. Gordus, J. P. Hasseman, G. Tsegaye, G. T. Holt, A. Hu, D. Walpita, R. Patel, J. J. Macklin, C. I. Bargmann, M. B. Ahrens, E. R. Schreiter, V. Jayaraman, L. L. Looger, K. Svoboda, D. S. Kim, Sensitive red protein calcium indicators for imaging neural activity. *eLife* **5**, e12727 (2016). doi:10.7554/eLife.12727 Medline

79. T. W. Dunn, Y. Mu, S. Narayan, O. Randlett, E. A. Naumann, C.-T. Yang, A. F. Schier, J. Freeman, F. Engert, M. B. Ahrens, Brain-wide mapping of neural activity controlling zebrafish exploratory locomotion. *eLife* **5**, e12741 (2016). doi:10.7554/eLife.12741 Medline

80. P. Antinucci, A. Dumitrescu, C. Deleuze, H. J. Morley, K. Leung, T. Hagley, F. Kubo, H. Baier, I. H. Bianco, C. Wyart, A calibrated optogenetic toolbox of stable zebrafish opsin lines. *eLife* **9**, e54937 (2020). doi:10.7554/eLife.54937 Medline

81. X. Yu, A. M. W. Taylor, J. Nagai, P. Golshani, C. J. Evans, G. Coppola, B. S. Khakh, Reducing astrocyte calcium signaling in vivo alters striatal microcircuits and causes repetitive behavior. *Neuron* **99**, 1170–1187.e9 (2018). doi:10.1016/j.neuron.2018.08.015 Medline

82. R. M. White, A. Sessa, C. Burke, T. Bowman, J. LeBlanc, C. Ceol, C. Bourque, M. Dovey, W. Goessling, C. E. Burns, L. I. Zon, Transparent adult zebrafish as a tool for in vivo transplantation analysis. *Cell Stem Cell* **2**, 183–189 (2008). doi:10.1016/j.stem.2007.11.002 Medline

83. A. Urasaki, K. Asakawa, K. Kawakami, Efficient transposition of the Tol2 transposable element from a single-copy donor in zebrafish. *Proc. Natl. Acad. Sci. U.S.A.* **105**, 19827–19832 (2008). doi:10.1073/pnas.0810380105 Medline

84. A. Bahl, F. Engert, Neural circuits for evidence accumulation and decision making in larval zebrafish. *Nat. Neurosci.* **23**, 94–102 (2020). doi:10.1038/s41593-019-0534-9 Medline

85. R.-W. Zhang, J.-L. Du, In vivo whole-cell patch-clamp recording in the zebrafish brain. *Methods Mol. Biol.* **1451**, 281–291 (2016). doi:10.1007/978-1-4939-3771-4_19 Medline

86. I. Shainer, E. Kuehn, E. Laurell, M. Al Kassar, N. Mokayes, S. Sherman, J. Larsch, M. Kunst, H. Baier, A single-cell resolution gene expression atlas of the larval zebrafish brain. *Sci. Adv.* **9**, eade9909 (2023). doi:10.1126/sciadv.ade9909 Medline

87. C. Leyden, T. Brüggemann, F. Debinski, C. A. Simacek, F. A. Dehmelt, A. B. Arrenberg, Efficacy of tricaine (MS-222) and hypothermia as anesthetic agents for blocking sensorimotor responses in larval zebrafish. *Front. Vet. Sci.* **9**, 864573 (2022). doi:10.3389/fvets.2022.864573 Medline

88. Y. Kumai, M. A. R. Ward, S. F. Perry, β-Adrenergic regulation of Na+ uptake by larval zebrafish Danio rerio in acidic and ion-poor environments. *Am. J. Physiol. Regul. Integr. Comp. Physiol.* **303**, R1031–R1041 (2012). doi:10.1152/ajpregu.00307.2012 Medline

89. S. Rahbar, W. Pan, M. G. Jonz, Purinergic and cholinergic drugs mediate hyperventilation in zebrafish: Evidence from a novel chemical screen. *PLOS ONE* **11**, e0154261 (2016). doi:10.1371/journal.pone.0154261 Medline

90. A. Bartoszek, A. Sumara, A. Kozub-Pędrak, A. Trzpil, A. Stachniuk, E. Fornal, Caffeine decreases topiramate levels in zebrafish larvae in a pentylenetetrazol-induced seizure model. *Int. J. Mol. Sci.* **25**, 3309 (2024). doi:10.3390/ijms25063309 Medline

91. A. M. Siebel, F. P. Menezes, K. M. Capiotti, L. W. Kist, I. da Costa Schaefer, J. Z. Frantz, M. R. Bogo, R. S. Da Silva, C. D. Bonan, Role of adenosine signaling on pentylenetetrazole-induced seizures in zebrafish. *Zebrafish* **12**, 127–136 (2015). doi:10.1089/zeb.2014.1004 Medline

92. L. Fontenas, T. G. Welsh, M. Piller, P. Coughenour, A. V. Gandhi, D. A. Prober, S. Kucenas, The neuromodulator adenosine regulates oligodendrocyte migration at motor exit point transition zones. *Cell Rep.* **27**, 115–128.e5 (2019). doi:10.1016/j.celrep.2019.03.013 Medline
